# Redox control and mechanisms of transmembrane signaling in CSS domain c-di-GMP phosphodiesterases that control biofilm formation in *Escherichia coli*

**DOI:** 10.1128/mbio.01325-26

**Published:** 2026-09-01

**Authors:** Katharina Pressler, Martin Lorkowski, Regine Hengge

**Affiliations:** 1 Institute of Biology/Microbiology, Humboldt-Universität zu Berlin 9373, Berlin, Germany; 2 Excellence Cluster Matters of Activity. Image Space Material, Humboldt-Universität zu Berlin 9373, Berlin, Germany; Universite de Geneve, Geneva, Switzerland

**Keywords:** biofilm, cellulose, curli, DsbA, redox regulation, second messenger

## Abstract

Bis-(3′,5′)-cyclic dimeric guanosine monophosphate (c-di-GMP) control of biofilm formation in *Escherichia coli* K-12 is balanced by multiple diguanylate cyclases (DGCs) and c-di-GMP-specific phosphodiesterases (PDEs). Five of the 13 PDEs feature a periplasmic CSS domain with two conserved cysteines, flanked by transmembrane (TM) regions, and an enzymatically active cytoplasmic EAL domain. One of these (PdeC) was previously shown to be redox-regulated by DsbA/DsbB-mediated disulfide bond (DSB) formation in the cysteine serine serine (CSS) domain. Comparing all five CSS domain PDEs, we found them to fall into two groups with similar biochemical features, resulting in different consequences for PDE activity. PdeB, PdeC, and PdeG are more active when lacking the periplasmic DSB, resulting in diminished biofilm formation, while PdeN and PdeD are active in their oxidized forms. Using PdeB and PdeN as prototypes for the two groups, not only the periplasmic DSB but also differently charged amino acid motifs close to the transmembrane (TM) domains and a putative additional DSB in PdeN were identified as important for transmembrane signaling. All these elements, including its stable structural DSB in the periplasm, which can form independently of DsbA, maintain PdeN in a rigid, proteolysis-resistant active conformation. By contrast, for PdeB, the more rigid DSB-containing conformation is inactive, with mutations in the stabilizing elements leading to a structurally less constrained, more active enzyme. Notably, low PdeN levels are post-transcriptionally upregulated at acidic pH, resulting in less biofilm formation. Overall, the five CSS domain PDEs enable *E. coli* to adapt to diverse environmental niches.

**IMPORTANCE:**

Sensing environmental cues and transmembrane signal transduction via membrane-embedded proteins is a process of key importance in all living cells. To investigate the molecular mechanisms involved, we performed a systematic functional comparison of the five CSS domain phosphodiesterases of *Escherichia coli*, which degrade the bacterial second messenger c-di-GMP in response to redox and other signals. With a sensory domain in the periplasm linked to a cytoplasmic enzymatic domain, these proteins represent minimal devices for transmembrane signaling. We demonstrate that these signal-transducing enzymes fall into two functional classes with a similar periplasmic redox biochemistry resulting in opposite states of cytoplasmic enzymatic activity. Several characteristic sequence elements convey redox and structural information in the periplasmic and transmembrane protein segments to their ability to dimerize into an enzymatically active form in the cytoplasm. Comparing the five enzymes also shows that evolution has played with these elements to facilitate adaptation to various environmental niches.

## INTRODUCTION

Bis-(3′,5′)-cyclic dimeric guanosine monophosphate (c-di-GMP) is a ubiquitous second messenger in bacterial cells with a pivotal influence on fundamental physiological processes, including general stress responses, virulence, and the transition to multicellularity, such as in biofilm formation (1, 2). Biofilms are communities of bacteria enveloped and held together by a protective extracellular matrix of biopolymers that can include secreted proteins, exopolysaccharides, and extracellular DNA (3). In *Escherichia coli* macrocolony biofilms, this extracellular matrix consists of amyloid curli fibers and phosphoethanolamine (pEtN) cellulose, which form a tightly entangled composite material that provides stability, cohesion, and elasticity to the biofilm (4–6). Biofilms are crucial in the etiology of chronic infections, as they confer increased bacterial tolerance against antibiotics and facilitate the evasion of host immune systems (7, 8).

As a key regulator of these processes, c-di-GMP is controlled by a dynamic interplay between its synthesis, mediated by diguanylate cyclases (DGC) carrying glycine, glycine, aspartic acid, glutamic acid, phenylalanine (GGDEF) domains, and its degradation, facilitated by specific phosphodiesterases (PDE) containing glutamic acid, alanine, leucine (EAL) or histidine, aspartic acid-glycine, tyrosine, proline (HD-GYP) domains (9). These enzymes can exist as cytoplasmic or membrane-associated variants and often incorporate N-terminal sensor domains, which effectively modulate their enzymatic activities including Per-Arnt-Sim (PAS), membrane-associated sensor (MASE), cyclase and histidine kinase-associated sensory extracellular (CHASE), gammaproteobacterial periplasmic sensor (GAPES), and, as addressed in this paper, CSS domains (named after a typically conserved and functionally essential cysteine-serine-serine motif) (10, 11). Nonetheless, our understanding of how sensory input mechanisms control the activities of these c-di-GMP-related enzymes has remained limited. Furthermore, numerous bacterial species possess multiple DGCs and PDEs with diverse sensor domains. Thus, *E. coli* K-12 shows a repertoire of 12 DGCs and 13 PDEs, most of which are not only expressed but also are enzymatically active at the same time (10, 12, 13). Notably, various commensal and pathogenic strains of *E. coli* harbor additional DGCs and PDEs, emphasizing the significance, diverse nature, and rapid evolutionary adaptation of c-di-GMP signaling across populations of even the same bacterial species (14).

In this paper, we analyze the widespread family of transmembrane c-di-GMP-degrading enzymes known as CSS domain PDEs that are characterized by two highly conserved cysteines—one of them in the name-giving cysteine-serine-serine motif—in their N-terminal periplasmic domain. This extracytoplasmic CSS domain is flanked by two transmembrane (TM) segments and directly followed by the cytoplasmic EAL domain with PDE activity. *E. coli* K-12 contains five members of this type of PDE, that is, PdeB, PdeC, PdeD, PdeG, and PdeN, with other enteric bacteria or pseudomonads possessing comparable numbers. One of the *E. coli* enzymes (PdeC) was previously shown to undergo regulatory free thiol/disulfide bond (DSB) transitions in response to the redox state of the periplasm (15). Genetic analyses as well as *in vitro* reconstitution in nanodiscs showed that DSB formation in the periplasmic CSS domain of PdeC, which *in vivo* is facilitated by the DsbA/DsbB system, reduces its PDE activity, whereas mutations eliminating either of the two cysteine residues activate PdeC, which leads to diminished biofilm matrix production. Activation of the free-thiol form of PdeC involves dimerization via the transmembrane segment following the CSS domain (TM2) (15).

In addition, the probably partially unfolded free-thiol form of the CSS domain of PdeC—obtained either by eliminating one or both cysteines by mutation or by treating cells with dithiothreitol (DTT)—is prone to proteolytic attack by the periplasmic proteases DegP and DegQ. This generates a TM2-EAL domain fragment, which can dimerize and shows activity as the complete free-thiol form of PdeC. Although the latter can switch back to the oxidized, less-active conformation, the processed TM2-EAL variant becomes inactivated by further degradation. Which of the inactivation pathways prevails will also depend on the availability of these two periplasmic heat-shock proteases, which, in turn, reflects a potential competition with other partially unfolded substrate proteins (15).

Why does *E. coli* K-12 possess five CSS domain PDEs? Does sharing a similar domain architecture mean that they all show the same biochemical reactivities and transmembrane signal transduction mechanism, or are there interesting molecular variations that may contribute to different physiological functions? Despite the common domain architecture, the primary amino acid sequences of the five CSS domains are remarkably variable except for the positions of the two conserved cysteines (one being in the CSS motif) and the overall domain lengths. In this study, we observed that while biochemical reactivities, such as DSB formation and proteolytic processibility, are similar, the five CSS domain PDEs fall into two subgroups, for which redox changes and concomitant structural changes in the CSS domain have even opposite functional consequences. Furthermore, we identify specifically located small amino acid regions of characteristically different charges in the CSS domains as well as an additional putative DSB bond in the TM2 region specifically of PdeN that are required for stabilizing CSS domain PDEs in either the active or the inactive conformation. Our data provide insight into the molecular mechanism of transmembrane signaling in these—with just two domains separated by the membrane—structurally minimal enzymes. Moreover, they suggest that structurally and biochemically very similar members of a single protein family can differentially adapt to distinct niches and/or integrate various environmental conditions.

## RESULTS

### The redox state of the periplasmic CSS domain oppositely affects the enzymatic activity of two subsets of the five CSS domain PDEs in *E. coli* K-12

Since the morphology of *E. coli* macrocolony biofilms depends on extracellular matrix production, which is, in turn, controlled by c-di-GMP, wrinkly macrocolony patterns are a sensitive indicator for the activities of DGCs and PDEs (2). An appropriate strain for such assays is *E. coli* K-12 AR3110, a direct derivative of the classical curli-producing but cellulose-negative laboratory strain W3110, in which cellulose biosynthesis was “repaired” by replacing the stop codon in *bcsQ* with a sense codon (as present in other cellulose-positive *E. coli* strains) (4). AR3110 synthesizes high levels of both curli fibers and pEtN cellulose, resulting in extremely flat large colonies that buckle up into high radial ridges and interspersed small wrinkles. Less matrix production results in smaller, yet higher colonies that exhibit small wrinkles only or are even completely unstructured. Congo red added to the agar plates stains both matrix components and serves as an additional highly sensitive matrix indicator (4, 16).

**Fig 1:**
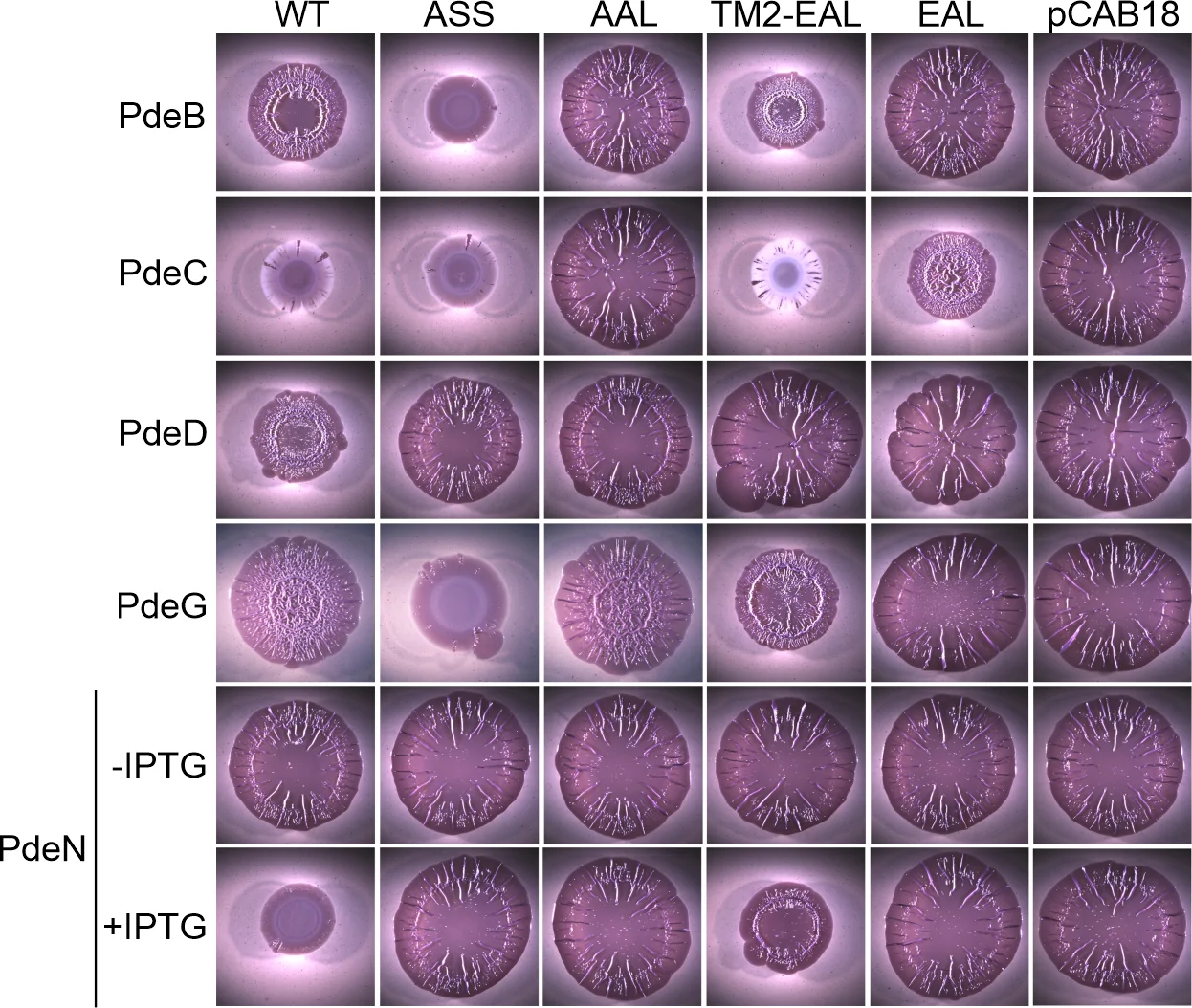
Role of the redox state of the CSS domain and the following TM2 segment in controlling PDE activity. Macrocolonies of *E. coli* K-12 strain AR3110 expressing the indicated PDE variants from the low-copy-number plasmid pCAB18 were grown on salt-free LB agar plates supplemented with the matrix dye Congo red for 5 days at 28°C. To compensate for very low protein levels, PdeN and its derivatives were additionally induced with 0.1 mM IPTG. WT, wild type; ASS, cysteine in the CSS motif substituted by alanine; AAL, E in the EAL motif substituted by alanine; TM2-EAL, lacking TM1 and the periplasmic CSS domain; and EAL, variant lacking the TM1, periplasmic domain, and the TM2. Four colony images in the “PdeB” row (WT, ASS, AAL, and pCAB18) were previously published (15) and are shown here again since they were generated as parts of the full-scale experiment comparing all CSS domain PDEs shown here.

**Fig 2:**
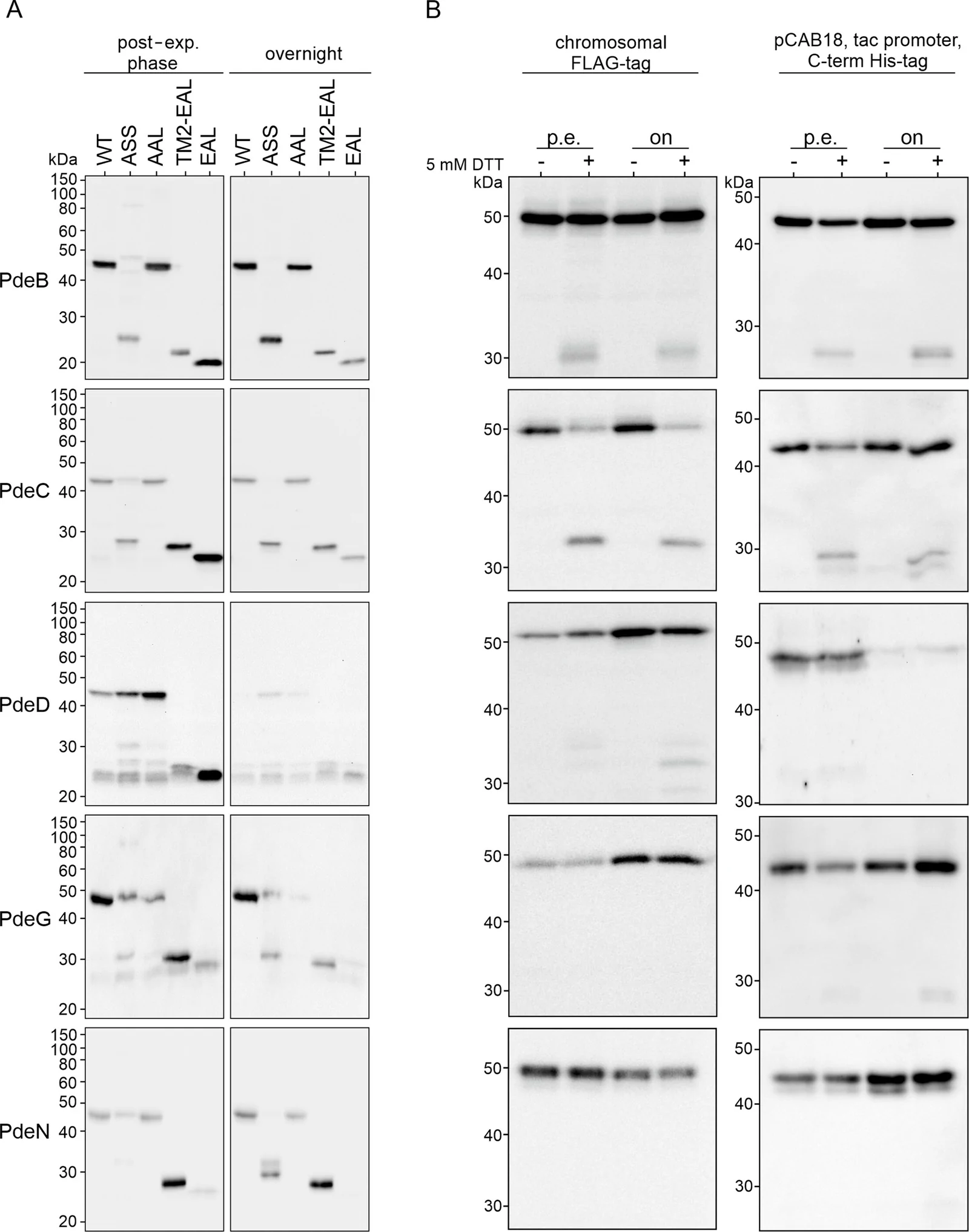
DSB formation in the CSS domain stabilizes against proteolysis. (**A**) Derivatives of W3110 expressing the C-terminal His6-tagged PDE variants from the low-copy-number plasmid pCAB18 were grown in liquid salt-free LB cultures. Samples were taken during the post-exponential growth phase (post-exponentially [p.e.]; OD_578_ approx. 2.5) and from overnight cultures at 28°C. (**B**) W3110 derivatives expressing the CSS domain PDEs either from the chromosomal genes (C-terminally tagged with a 3× FLAG-tag) or from plasmid pCAB18 (with the His6-tag) were grown as in (**A**), but in the presence or absence of 5 mM DTT, and the samples were taken from p.e. growing or overnight liquid cultures (grown at 28°C). CSS domain PDEs and their proteolytic fragments were visualized by SDS-PAGE and immunoblotting using antibodies against the respective tags.

To test the activity of the five CSS domain PDEs by using macrocolony morphology as an initial readout, we cloned their genes under the control of the *tac* promoter (*P_tac_*) onto a low-copy-number expression plasmid (pCAB18). In addition, point mutations in the coding sequences of the five PDE genes were introduced to either replace the conserved cysteine in the CSS motif by alanine (“ASS”), thereby preventing periplasmic DSB formation, or to replace the active site glutamate in the EAL motif by alanine (“AAL”) in order to disrupt PDE activity (15, 17). While a majority of PDEs feature the name-giving EAL motif, some CSS domain PDEs (as well as various other PDEs) slightly deviate from this motif. PdeN and PdeG feature the second common EVL motif, while PdeD shows EIL, but these are known to be active variants (18). For formal reasons of functional similarity, derivatives of these CSS domain PDEs with the E replaced by A were all termed here “AAL” variants. Furthermore, N-terminal deletion variants consisting of just the transmembrane domain 2 (TM2) linked to the EAL domain (“TM2-EAL“), as well as of the EAL domain alone (“EAL“), were constructed for all five CSS domain PDEs. All these protein variants were expressed within a range similar to that of the wild-type CSS domain PDEs (see Fig. 1 and 2).

The comparison of macrocolony biofilm morphology of strain AR3110 expressing these variants of the CSS domain PDEs (Fig. 1) revealed two functional subtypes of these PDEs that are characterized by opposite behavior when wild-type variants were compared to ASS variants. This was most clearly apparent for PdeB, where the PdeB^ASS^ variant was more active than PdeB^WT^ (resulting in smaller non-wrinkled colonies of the former), and for PdeN, for which this pattern was exactly opposite (but where detection required IPTG induction of P*_tac_* because of the very low basal expression of PdeN that is further addressed below; note that the expression of the other CSS domain PDEs just made use of the non-induced basal or “leaky” expression from P*_tac_* in order to remain in a physiological range of expression). The other members of the “PdeB group” were PdeC and PdeG. Notably, all PdeC variants seemed to have higher activity than their counterparts derived from the other four CSS domain PDEs, and therefore, moderate overproduction (from the plasmid used here) tended to blur the also previously observed difference between the PdeC^WT^ and PdeC^ASS^ (15), while PdeG seemed to have generally very low activity (with PdeG^WT^ not being different from the inactive PdeG^AAL^; Fig. 1). On the other hand, PdeD fell into the “PdeN group”, that is, PdeD^WT^, was slightly more active than PdeD^ASS^, but generally, the PdeD variants showed very low activity only (Fig. 1). Notably, these effects were due to modulation of the PDE activity as confirmed by the disappearance of these phenotypes with the PDE variants carrying inactive EAL domains (AAL variants), which exhibited a biofilm matrix morphology comparable to AR3110 with just the empty vector (Fig. 1).

Overall, these data thus indicate that CSS domain PDEs fall into two groups, in which the periplasmic DSB contributes in an opposite manner to activation of the PDE activity of the cytoplasmic EAL domain. The “PdeB group” (also including PdeC and PdeG) is more active in the absence of the DSB in the CSS domain, whereas the “PdeN group” (also including PdeD) shows higher activity with the CSS domain in the oxidized form.

An interesting question was therefore also how the complete absence of the redox-controllable CSS domain affects the activities of these two groups of PDEs. As previously reported (15), the TM2-EAL fragment of PdeC expressed from the low-copy-number plasmid showed high enzymatic activity (the macrocolonies were even hardly stained with Congo red; Fig. 1). Similar minimal constructs, which completely lack the respective TM1 and the CSS domains, were found to be active not only for PdeB and, to a lesser extent, for PdeG but also for PdeN (IPTG-induced), while for the PdeD derivative, no activity was observed (but even PdeD^WT^ had very low activity only; Fig. 1). For those four TM2-EAL fragments that showed activity, the corresponding minimal EAL domains alone were inactive or, in the case of the EAL fragment of PdeC, only very weakly active (Fig. 1). Overall, this suggests that TM2 plays a similar role as previously shown for PdeC, namely to allow or to promote the dimerization of the EAL domain (15), in the activation mechanism in both groups of CSS domain PDEs. What differentiates the “PdeB group“ and “PdeN group” is thus the role of the periplasmic DSB in the CSS domain in transmembrane signaling that either reduces or promotes PDE activity, respectively.

### Role of the redox state of the periplasmic CSS domain in proteolysis of the five CSS domain PDEs in *E. coli* K-12

In the previous study, the PdeC^ASS^ variant, which is unable to form the DSB in the periplasmic CSS domain, was found to be a target for proteolytic degradation by the periplasmic proteases DegP and DegQ (15). Proteolytic degradation patterns were therefore analyzed here by immunoblotting of all five CSS domain PDEs and their mutant derivatives (C-terminally His-tagged and again expressed from the low-copy-number plasmid; Fig. 2A). During the post-exponential growth phase, a stable single band could be detected for the wild-type variants of PdeB, PdeC, PdeG, and PdeN. Only PdeD^WT^ also generated some smaller fragments, which points toward some protein instability that became more significant in overnight samples, where hardly any full-size band of PdeD^WT^ could be detected. By contrast, the ASS variants of all CSS domain PDEs showed significant degradation to a fragment of typically 26–28 kDa (Fig. 2A), just as previously described for PdeC (15). In the case of PdeN^ASS^, this proteolytic fragment became visible only after overnight growth.

Furthermore, for PdeB^WT^, PdeC^WT^, and PdeD^WT^, the formation of this proteolytic fragment could also be induced by the addition of dithiothreitol (DTT; a reducing reagent that breaks down DSBs) to the growing cultures, no matter whether single copy chromosomally FLAG-tagged or plasmid-encoded HIS-tagged constructs were used (Fig. 2B). Only PdeN^WT^ was entirely insensitive to such reducing treatment, no matter whether expressed from the chromosomal gene or overexpressed from the plasmid (IPTG-induced) or whether detected using a His-tag or a FLAG-tag. Also for PdeG, only traces of the proteolytic band could be detected in late stationary phase (overnight) when PdeG^WT^ was overproduced (Fig. 2B). Whenever observed, the proteolytic fragments obtained from the ASS variants of the CSS domain PDEs were slightly larger than the corresponding TM2-EAL fragments (Fig. 2A), indicating processing to the proteolytic fragment within the periplasmic CSS domain.

Overall, these data indicate that the absence of the periplasmic DSB—either due to the ASS mutation in the CSS domain or as a consequence of DTT treatment—probably partially opens up the conformation of the CSS domain, which results in periplasmic proteolysis breaking down the full-size CSS domain PDEs to protein fragments typically slightly larger than the TM2-EAL fragment obtained by direct cloning. However, the degree to which this proteolytic processing could be observed for the five CSS domain PDEs was very different. While it occurred readily with PdeB and PdeC and still to some degree with PdeD and PdeG, it could not be detected for PdeN under all conditions tested. This suggests that the periplasmic DSB in PdeN is more stable than in the other CSS domain PDEs, which points toward a structural DSB rather than a reversible regulatory DSB.

### Roles for DsbA in periplasmic DSB formation and for DegP/DegQ in proteolysis of the five CSS domain PDEs

**Fig 3:**
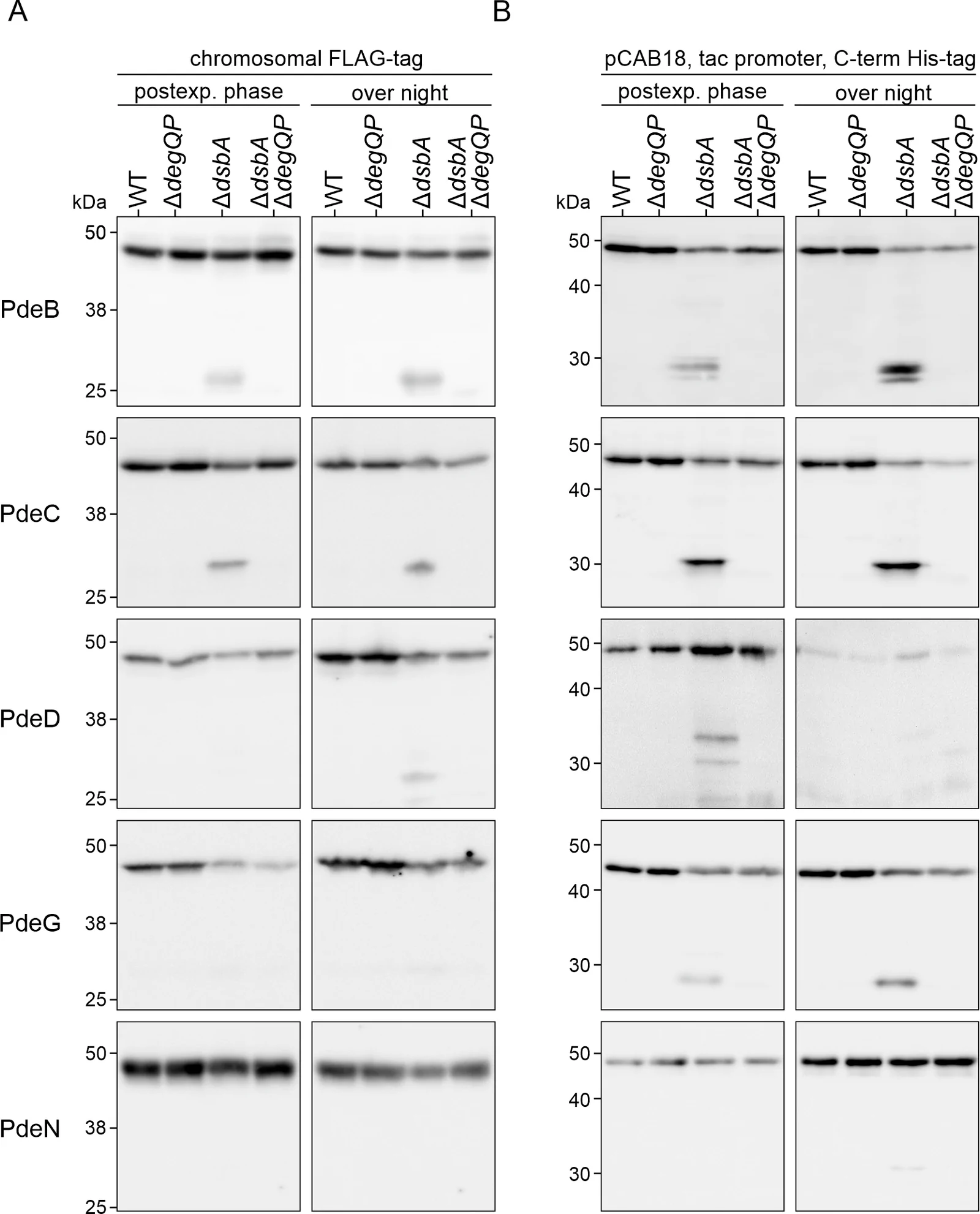
Roles of DsbA in DSB formation and of DegP/DegQ in proteolysis. Derivatives of W3110 (carrying mutations in *degP*/*degQ* and/or *dsbA* as indicated) expressing CSS domain PDEs either (**A**) with a chromosomal 3× FLAG-tag or (**B**) from the low-copy-number plasmid pCAB18 with a His6-tag were grown to post-exponential phase (OD_578_ approx. 2.5) and overnight in liquid salt-free LB culture at 28°C. CSS domain PDEs and their proteolytic fragments were visualized by SDS-PAGE and immunoblotting using antibodies against the respective tags.

The DsbA/DsbB system consists of the redox-active enzymes primarily responsible for promoting periplasmic DSB formation in *E. coli* (19). It is also involved in DSB formation in the CSS domain of PdeC, as evidenced by *dsbA* or *dsbB* mutants being more prone to generating the proteolytic fragment of PdeC (15). Hence, we investigated whether the other CSS domain PDEs follow the same principle. Immunoblot analyses were performed using the wild-type variants of all five CSS domain PDEs, either chromosomally FLAG-tagged or overexpressed with a His-tag from pCAB18, in a *dsbA* deletion mutant (Fig. 3A and B). As previously shown for PdeC, where the periplasmic DSB is promoted by the DsbA/DsbB system, a degradation fragment like that in the DSB-free ASS variants was also observed for PdeB and PdeD in the *dsbA* deletion mutant (Fig. 3A and B). For PdeG, the corresponding proteolytic fragment was only found when the protein was overproduced (Fig. 3B). These findings suggest that the DsbA/DsbB system facilitates DSB formation in the periplasmic loops of these four CSS domains PDEs and thereby enhances their structural stability and protects them from proteolysis.

Interestingly, PdeN again behaves differently from the other four CSS domain PDEs. Its protein stability did not significantly change in the *dsbA* knockout background, that is, a proteolytic fragment was not observed, no matter whether the protein was expressed chromosomally with a C-terminal FLAG-tag or overexpressed with a C-terminal His-tag from the plasmid (Fig. 3A and B). Only after overnight growth and with PdeN overexpressed, a faint trace of a proteolytic fragment may be detectable in the *dsbA* mutant (Fig. 3B). We conclude that—in contrast to the other four CSS domain PDEs—DSB formation and folding of PdeN into a stable proteolysis-resistant conformation is largely independent of the DsbA/DsbB system.

The proteases that play a key role in the degradation of the CSS domain PDE variants were identified as DegQ and DegP, since the proteolytic fragments appearing in a *dsbA* null mutant vanished again upon additionally deleting the genes encoding these two periplasmic proteases (Fig. 3A and B). However, just as previously observed for PdeC (15), also for the other CSS domain PDEs, where proteolytic processing occurs (PdeB, PdeD, and PdeG), DegQ/DegP-mediated degradation was not required for enzymatic activity since macrocolony morphologies were not significantly different when DegQ and DegP were either present or absent (Fig. S1). Consistent with PdeN not being degraded, mutating *degP* and *degQ* also had no effect on PdeN activity, as apparent from macrocolony morphologies (Fig. S1).

Overall, our data reveal a redox state-dependent proteolysis of PdeB, PdeC, PdeD, and PdeG by DegP/DegQ, with the free thiol forms (present either in the ASS variants or in the absence of DsbA/B) seemingly being less stably folded within the CSS domain and thus more accessible to partial degradation by DegQ/DegP. However, rather than being required for enzymatic activation, this proteolysis is a first step toward removal of these proteins. Also here, PdeN does not follow this principle. Its highly stable periplasmic DSB, which forms even in the absence of DsbA/B, contributes to a tightly folded conformation not targeted by DegP/DegQ-mediated proteolysis at all (Fig. 3). Even in the absence of its DSB (in PdeN^ASS^), it seems to retain a relatively stably folded conformation not efficiently attacked by these proteases (Fig. 2).

### The opposite effect of the periplasmic DSB on cytoplasmic PDE activity of PdeB and PdeN is associated with specific differentially charged amino acid patterns in the periplasmic CSS domain

**Fig 4:**
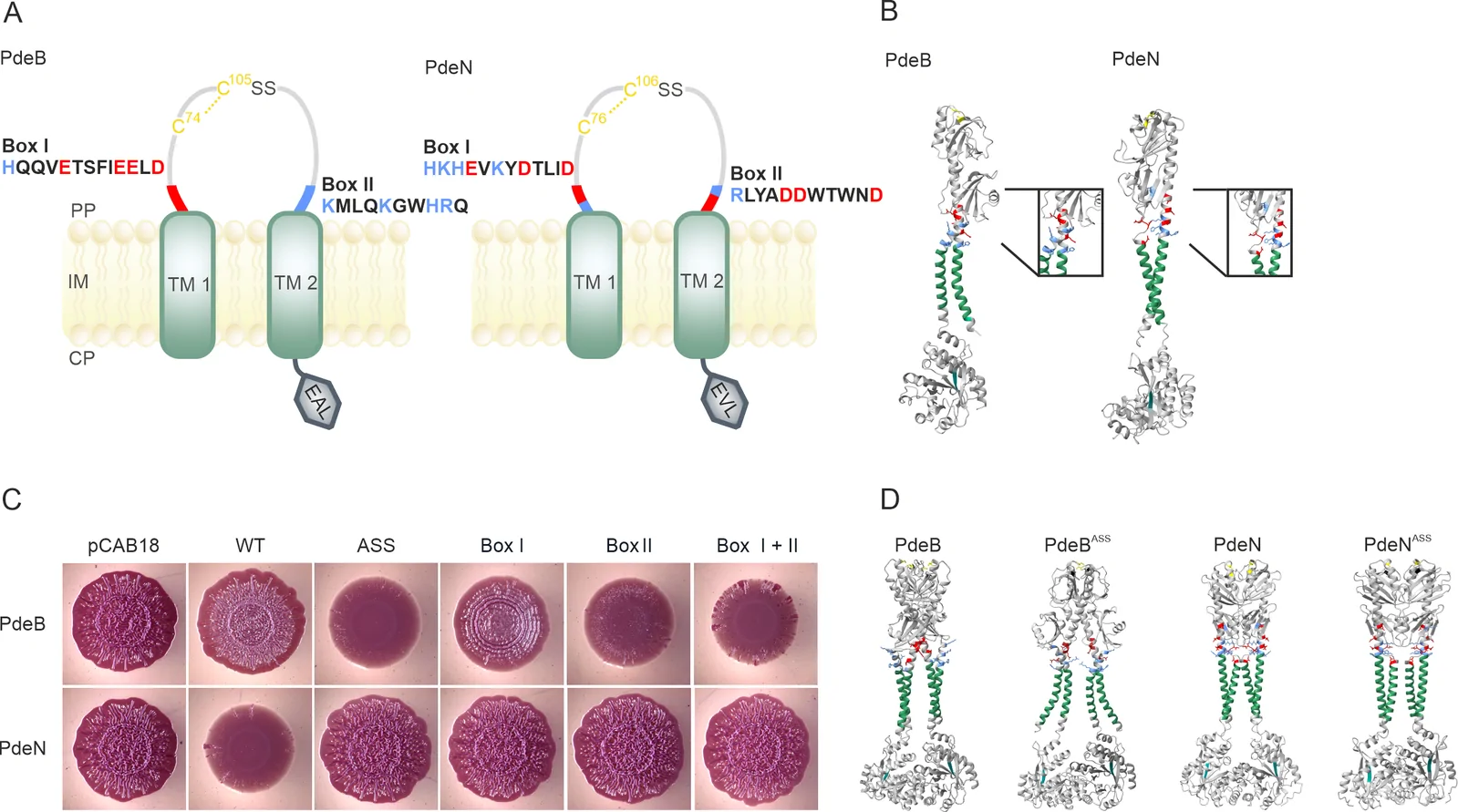
Highly charged amino acid segments located on the periplasmic side next to the two transmembrane domains have an impact on PDE activity. (**A**) Graphical illustration of PdeB and PdeN with TM1 and TM2 shown in green and directly adjacent amino acid segments (box I and box II) colored according to the distribution of charge. The corresponding sequences are shown next to the boxes, with blue and red highlighting positively and negatively charged amino acids, respectively. The loop connecting TM1 and TM2 represents the periplasmic domain, with the cysteines forming the DSB (including the one in the CSS motif) highlighted in yellow. The cytoplasmic EAL domain is not drawn to scale. (**B**) AlphaFold2 model of the PdeB and PdeN monomers with a close-up of the part featuring the charged amino acid box I and box II highlighted in black rectangles. (**C**) Macrocolonies of *E. coli* K-12 strain AR3110 expressing the indicated variants for PdeB and PdeN from the low-copy-number plasmid pCAB18 were grown on salt-free LB agar plates (also containing Congo red) for 5 days at 28°C (nomenclature of mutant variants of PdeB and PdeN: PdeB^BoxI^ stands for PdeB with its box I replaced by box I of PdeN, etc.). PdeN versions were induced by adding 0.1 mM IPTG to the cultures. (**D**) Dimer models of PdeB^WT^, PdeB^ASS^, and PdeN^WT^ were generated with AlphaFold2. Monomer (**B**) and dimer models (**D**) displayed the protein structure of PDEs in the same color code as shown in A and were depicted using ChimeraX 1.5.

The data presented above point toward two subtypes of CSS domain PDEs: PdeB, PdeC, and PdeG are more active in their free-thiol forms, whereas PdeD and PdeN are more active in their periplasmically oxidized DSB forms. In order to further elucidate the underlying mechanistic differences, which may help reveal the mechanism of transmembrane signal transduction in CSS domain PDEs in general, we focused on the two most active CSS domain PDEs, which represent these two groups most clearly, that is, PdeB and PdeN. Apart from the common domain architecture and structural motifs—the two TM regions, the conserved cysteines in the periplasm and the cytoplasmic catalytic EAL domain (Fig. S2A)—the primary sequences of these two membrane-associated enzymes are quite diverse (Fig. S2B). Nevertheless, a comparison of PdeB and PdeN sequences at corresponding positions revealed interesting patterns. One is a patch of significantly differentially charged amino acids on the periplasmic sides just adjacent to their respective TM1 and TM2 regions (Fig. 4A). In PdeB, just following TM1, there is a sequence of predominantly negatively charged amino acids (His^32^–Asp^44^; further referred to as “box I”), while preceding TM2 (Lys^235^–Gln^244^; further referred to as “box II”), there is an abundance of positively charged amino acids (Fig. 4A; Fig. S2B). According to the “positive-inside rule” next to TM regions (20), this positively charged box II is highly unusual for periplasmic loop proteins. A somewhat different charge pattern is present in PdeN at the corresponding positions. Here, the box I region of PdeN (Lys^38^–Asp^49^) displays a mixture of amino acid charges, with a gradient consisting of positively charged amino acids close to the membrane (again violating the “positive-inside rule”) toward negatively charged amino acids pointing more into the periplasm (Fig. 4A; Fig. S2B). Also, the PdeN box II (Arg^225^–Asp^235^) shows a gradient of charge (positive toward negative just adjacent to TM2).

By potentially interacting via electrostatic interactions, these charged boxes I and II could influence protein conformations, and given the role of more or less tight folding of the periplasmic CSS domains in controlling transmembrane signaling, these boxes may be involved in the proper functioning of these CSS domain PDEs. For PdeB, an AlphaFold2 model (Fig. 4B) suggested with a high level of confidence (Fig. S2A) that the charged amino acid residues of both boxes do not point toward each other, but rather in opposite directions, as seen in the close-up (depicted by the black rectangular box in Fig. 4B). Thus, it seems unlikely that they intercalate via electrostatic forces. However, the negatively charged residues of box I and the positively charged residues of box II in PdeN are confidently arranged to fit to one another, suggesting that they contribute to maintaining PdeN in a stable conformation (Fig. 4B; Fig. S2A).

In order to test experimentally whether boxes I and II are relevant for enzymatic activity, we exchanged either box I, box II, or both boxes simultaneously between PdeB and PdeN and investigated the impact of these mutations on macrocolony morphology as a readout for PDE activity (with controlling for similar protein expression levels as shown in Fig. S3). Replacing box I of PdeB (mostly negatively charged) by box I of PdeN (showing a mixed charge pattern) altered macrocolony morphology to a pattern of concentric rings (Fig. 4C), which is typical for curli production remaining high and pEtN-cellulose being mostly absent (21). This is an indication of moderately increased PDE activity of this PdeB^BoxI^ variant, which affects cellulose biosynthesis more strongly probably because PdeB and cellulose synthase are both membrane-located and cellulose synthase, which has the lowest affinity for c-di-GMP of all known c-di-GMP targets in *E. coli* (22), requires high c-di-GMP production by a co-localized DGC undisturbed by nearby PDEs (13, 23, 24). Replacing box II or both boxes I and II in PdeB by the respective boxes of PdeN had a more drastically activating effect, with these variants (PdeB^BoxII^ and PdeB^BoxI+I^) essentially showing the same phenotype as PdeB^ASS^, which reflects strong PDE activation to the point that macrocolonies remained unstructured (Fig. 4C). Overall, all changes in box I and/or box II of PdeB led to a more or less strong *gain* in PDE activity, suggesting that its box I and box II both contribute to stabilize the inactive conformation of PdeB, although they are not predicted to directly interact with each other via electrostatic interaction. While just an algorithm-based model, the AlphaFold2 dimeric structures of the PdeB^WT^ and PdeB^ASS^ supported this interpretation. Eliminating the DSB in the periplasmic loop of PdeB (PdeB^ASS^ as shown in Fig. 4D) results in the prediction of a more open or flexible dimeric structure, which may allow the EAL domains to form the catalytically active dimer.

Contrary to that, PdeN *lost* its activity, no matter which box(es) were replaced by the corresponding boxes of PdeB, resulting in a wrinkly macrocolony phenotype similar to that of the empty vector control (Fig. 4C). This is fully consistent with PdeN being active in a highly structured or even rigid conformation that is not only stabilized by its periplasmic DSB but also by its boxes I and II. Accordingly, the active dimeric conformation of PdeN^WT^ modeled by AlphaFold2 seems a solid structure stabilized by its periplasmic DSB as well as by potential interactions in the box I and box II regions (Fig. 4D).

### A glycine-rich patch and two cysteines specifically in the TM regions of PdeN are required for PDE activity

**Fig 5:**
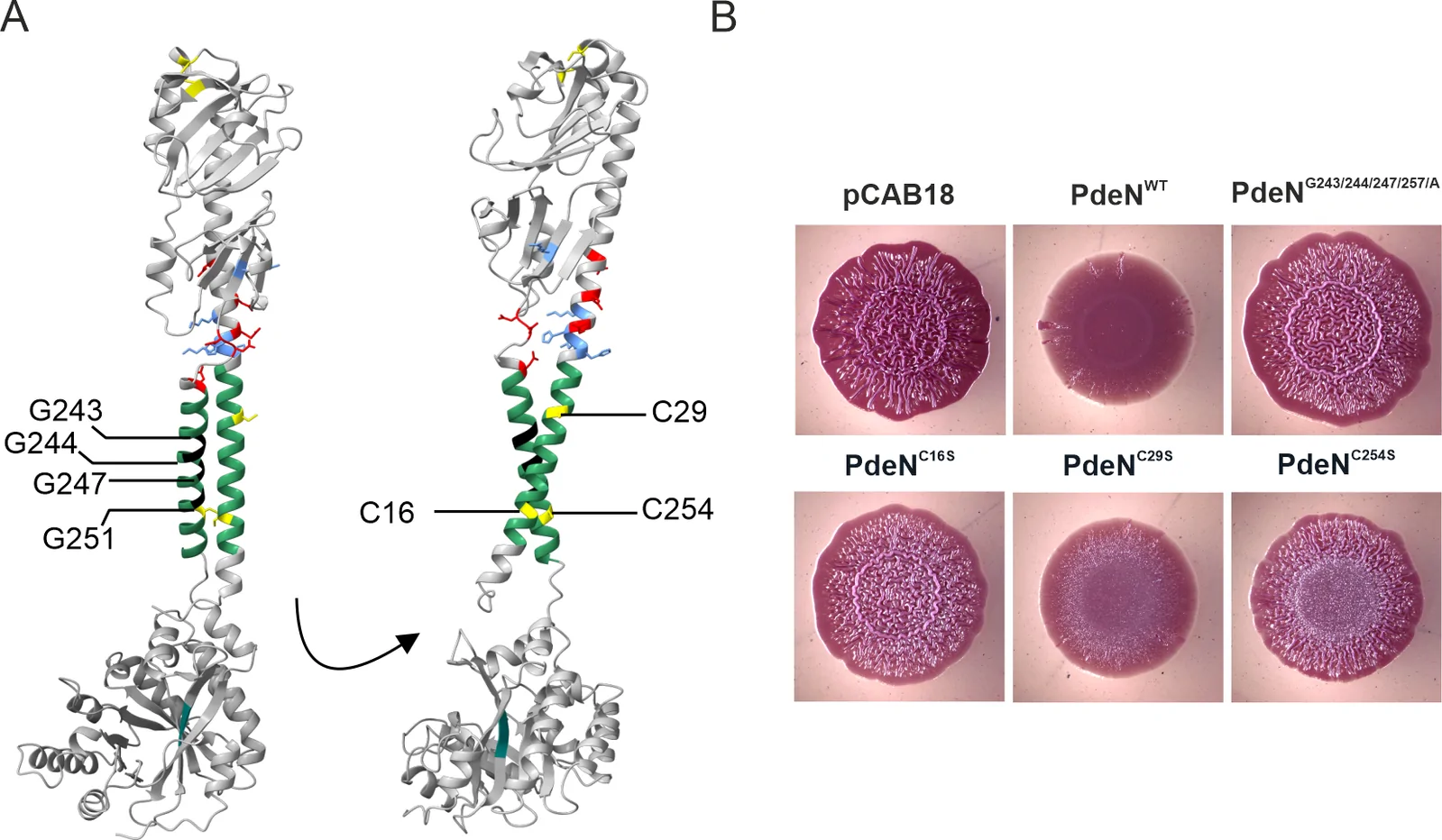
Additional amino acid motifs in PdeN relevant for PDE activity. (**A**) In the AlphaFold 2 model of PdeN^WT^, an array of glycine residues located in TM2 (left) and three cysteine residues (right) are labeled. (**B**) Macrocolonies of *E. coli* K-12 strain AR3110 expressing the PdeN variants with the indicated amino acid substitutions from the low-copy-number plasmid pCAB18 were grown on salt-free LB agar plates (also containing Congo red and 0.1 mM IPTG) for 5 days at 28°C. The colony image showing the empty vector control (pCAB18) is identical to the respective control colony image shown in Fig. 4, since all colonies shown in Fig. 4 and 5 were obtained as parts of the same experiment.

In addition to the mixed-charged boxes I and II and the conserved DSB in the CSS domain, PdeN displays two more interesting details that may influence its structure and thus enzymatic activity. Unlike in other CSS domain PDEs, the TM2 of PdeN contains four glycines, two of them in a GXXXG motif (G^247^XXXG^251^; Fig. S2B), that could be important for dimerization and/or introducing a slight kink in the TM2 domain (25, 26) to hold it in an appropriate orientation for full activation of the enzyme (Fig. 5A). Indeed, the substitution of the glycines (G^243/244/247/251^) by alanines eliminated PdeN activity as shown by strong macrocolony wrinkling, that is, increased biofilm matrix formation (Fig. 5B).

Furthermore, PdeN harbors three additional cysteines located in the TM regions besides the two conserved ones in the periplasm. In the AlphaFold2 model, Cys^16^ in TM1 and Cys^254^ in TM2 appear to be close enough to form an intramolecular DSB between the transmembrane domains (Fig. 5A). Substitution of Cys^16^ or Cys^254^ to serine resulted in reduced PdeN activity as indicated by wrinkly macrocolonies (Fig. 5B). The substitution of Cys^29^ to Ser in PdeN had hardly any effect (Fig. 5B). All these results together indicate that PdeN activity depends on a rigid conformation, which is stabilized not only by the periplasmic DSB in the CSS domain but also by additional structural peculiarities, which include an array of glycines in TM2 and a unique DSB (C^16^–C^254^) that connects TM1 and TM2. This is in pronounced contrast to PdeB, which is active with its CSS domain in the more flexible free thiol conformation. Consistent with this, PdeN can be *inactivated* by mutating any of these stabilizing elements (Fig. 4 and 5), whereas PdeB is *activated* by any amino acid changes that promote structural flexibility (Fig. 4).

### Redox sensitivity of *in vitro* activities of PdeB and PdeN reconstituted into nanodiscs

**Fig 6:**
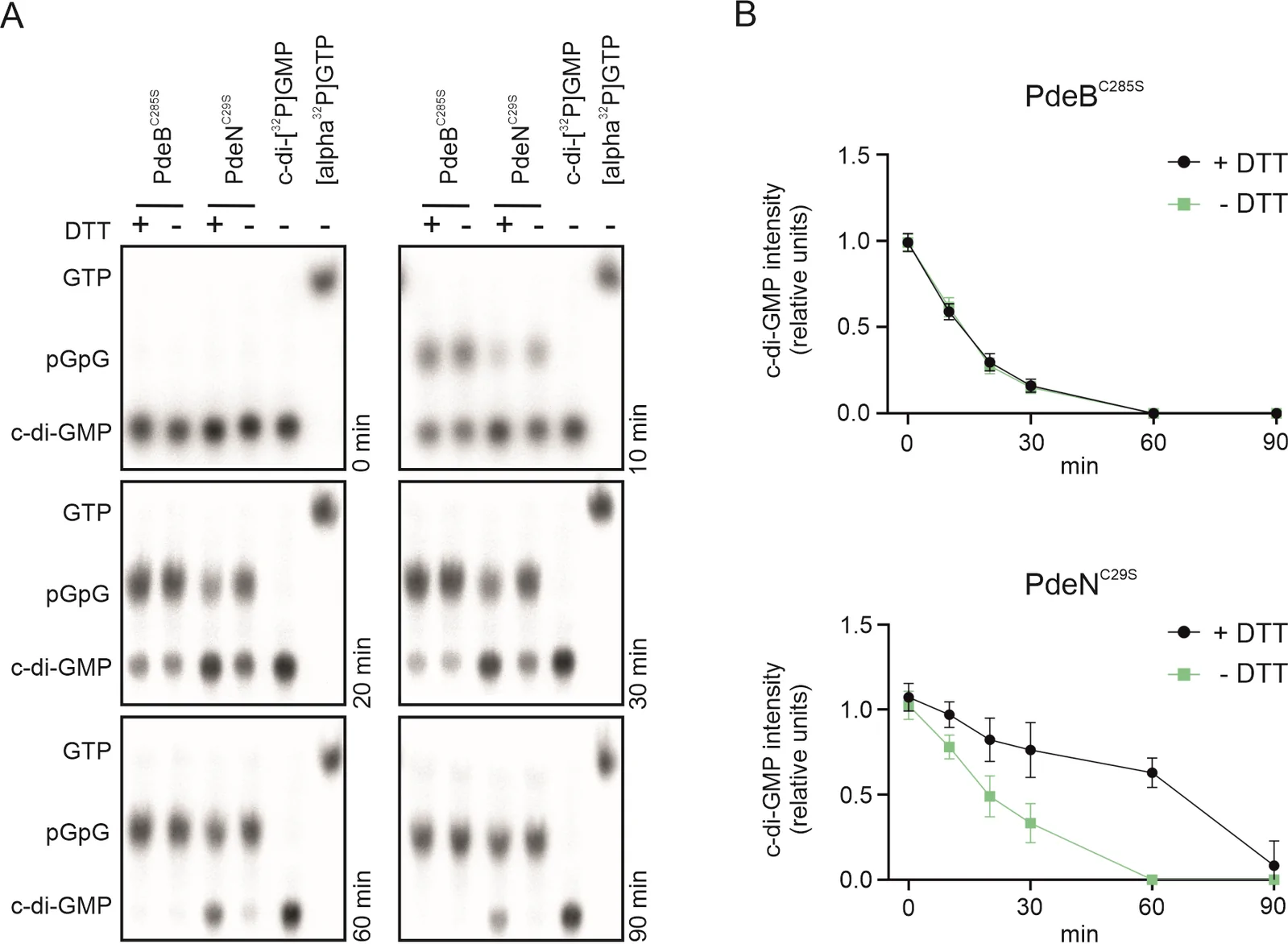
Phosphodiesterase activity of purified PdeB^C285S^ and PdeN^C29S^ reconstituted into nanodiscs. (**A**) Detergent-solubilized and purified PdeB^C285S^ and PdeN^C29S^ were reconstituted into nanodiscs and assayed for PDE activity using [α-^32^P]c-di-GMP in the absence or presence of 5 mM DTT. Samples were taken at the times indicated (from top to bottom), and the remaining radiolabeled substrate c-di-GMP and the enzymatic product 5′-pGpG were detected by thin-layer chromatography and autoradiography. As a control for GTP, [α-^32^P]GTP was used. The experiment was performed three times independently, with a representative example shown here. (**B**) Quantification of radiolabeled c-di-GMP spots as visualized in panel **A** is shown as the enzymatic turnover of c-di-GMP by purified and nanodisc-reconstituted PdeB^C285S^ (top) and PdeN^C29S^ (bottom) in the absence (green squares) and presence of DTT (black dots). Error bars indicate the median with an interquartile range of three independent experiments.

Nanodiscs consist of self-assembled discoidal phospholipid bilayers that are stabilized by a membrane scaffold protein that is wrapped around the bilayer, in which a membrane protein can be incorporated. Thus, they mimic a native membrane environment for the protein (27–29). For reconstitution into nanodiscs, we used PdeB and PdeN variants, in which any additional cysteines (beyond those required for functional DSB formation) were substituted by serines to prevent non-canonical and potentially inhibitory DSB formation during the protein purification process under aerobic conditions. PDE activity in the absence or presence of DTT—corresponding to DSB or free-thiol forms, respectively—was thus determined with nanodiscs containing reconstituted PdeB^C285S^ and PdeN^C29S^ using thin-layer chromatography to detect the conversion of radiolabeled c-di-GMP into the linear pGpG product (Fig. 6A, with quantification shown in Fig. 6B).

Treating reconstituted PdeN^C29S^ with DTT indeed reduced its enzymatic PDE activity, consistent with PdeN being active in its oxidized wild-type form (Fig. 6A and B). In the case of PdeB^C285S^, the addition of DTT did not have an effect on the enzymatic activity *in vitro* (Fig. 6A and B). In fact, the activity observed for reconstituted PdeB^C285S^ was already relatively high (compared to PdeN^C29S^) in the absence of DTT, which may have interfered with further stimulation. This high activity already in the absence of DTT could be an indication that *in vitro* DSB formation of isolated PdeB^C285S^ is inefficient, consistent with DSB formation *in vivo* being dependent on the DsbA/B system (Fig. 3). In addition, the single C285S substitution in the EAL domain of PdeB may also somewhat disturb activation, since it seems to reduce PDE activity slightly when also introduced in the otherwise highly active PdeB^ASS^ variant *in vivo* (Fig. S4).

### PdeN is post-transcriptionally induced and controls biofilm formation at low pH

Our observation that PdeN is active in a structurally rigid form, which depends on the stable DsbA-independent DSB in the CSS domain as well as other sequence features in its TM regions, suggests that the enzymatic activity of PdeN is not directly regulated. Rather, PdeN could be upregulated at the level of expression under some specific conditions. Consistent with a very low basal expression level, we had to add an inducer (IPTG) to obtain immunoblot-detectable protein levels and physiological effects of PdeN expressed from a plasmid, whereas the other CSS domain PDEs expressed from similar constructs did not require induction in order to show clear expression and activity (Fig. 1 and 2). The fact that the expression of all these constructs was under the control of the same ectopic promoter (p*_tac_*) suggested some negative post-transcriptional control of PdeN.

**Fig 7:**
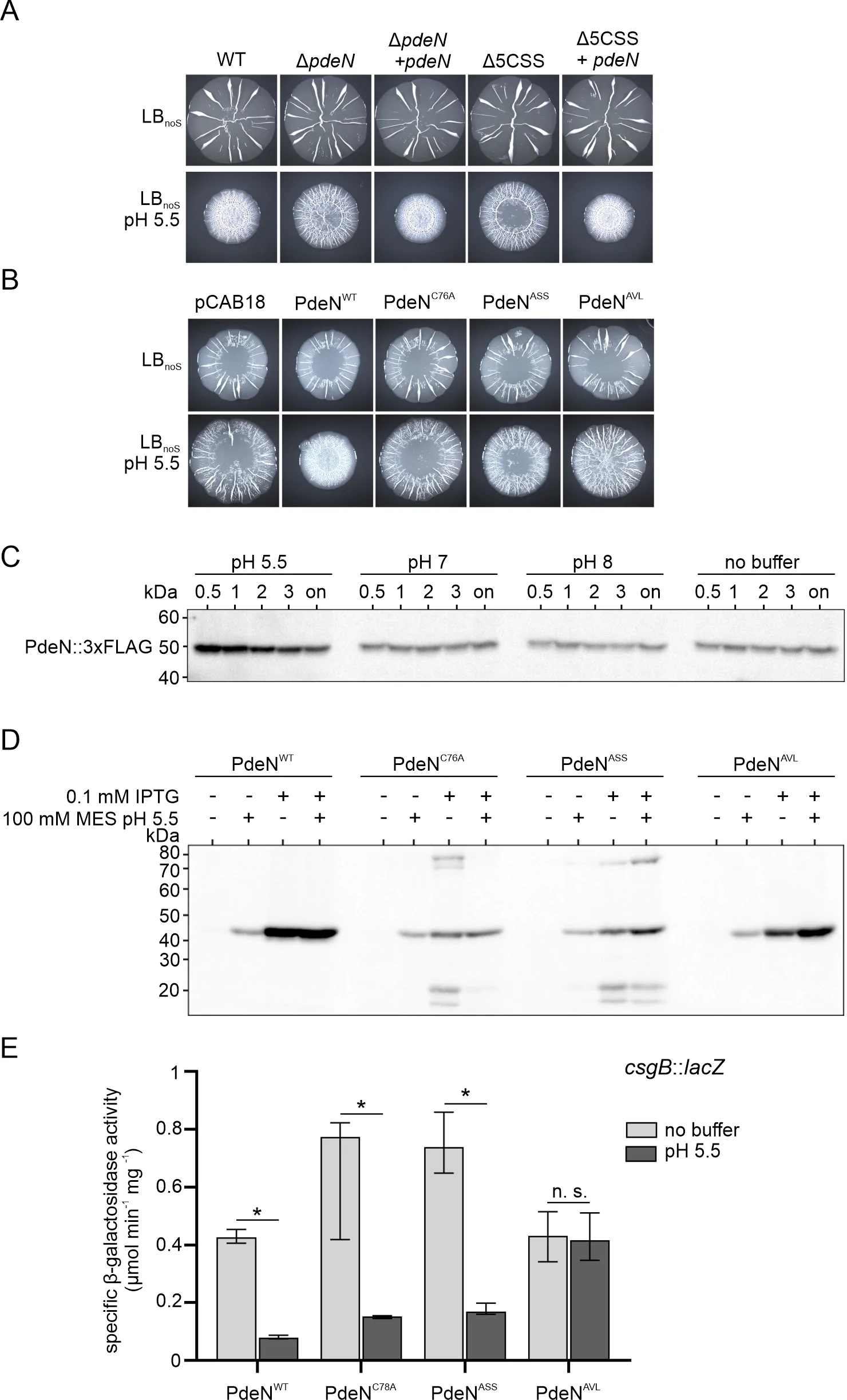
PdeN plays a physiological role in biofilm formation and is induced during growth at low pH. (**A**) Macrocolonies of *E. coli* K-12 strain AR3110 (WT) and the indicated mutant derivatives were grown for 5 days at 28°C on non-buffered salt-free LB agar plates (upper panel), and the same medium was adjusted to pH 5.5 (by adding MES; lower panel). (**B**) Macrocolonies of *E. coli* K-12 strain AR3110 expressing the indicated *pdeN* variants from the low-copy-number plasmid pCAB18 were grown under the same conditions as in panel **A**. (**C**) A derivative of strain W3110 expressing PdeN chromosomally with a C-terminal 3× FLAG-tag was grown in liquid salt-free LB at 28°C, with the growth medium adjusted to pH 5.5 (100 mM MES added), pH 7, or pH 8 (100 mM MOPS added) or without buffering. Samples were taken at OD_578_ of 0.5, 1, 2, and 3 and from overnight cultures, and PdeN was visualized by SDS-PAGE and immunoblotting using antibodies against the 3× FLAG-tag. (**D**) Derivatives of strain W3110 expressing the indicated C-terminally His6-tagged PdeN variants from pCAB18 were grown at 28°C in LBnoS also containing 0.1 mM IPTG and/or 100 mM MES as indicated. Samples were taken after overnight growth, and the PdeN variants as well as degradation fragments and stable dimers (obtained with variants containing one cysteine only in the CSS domain) were visualized by SDS-PAGE and immunoblotting using antibodies against the 3× FLAG-tag. (**E**) Derivatives of strain W3110 carrying a single-copy *csgB::lacZ* reporter fusion and expressing the indicated PdeN variants from pCAB18 were grown overnight at 28°C in salt-free LB (also containing 0.1 mM IPTG) that was either unbuffered or contained 100 mM MES to adjust the pH to 5.5, and specific β-galactosidase activities were determined. Error bars indicate the median with an interquartile range of three independent experiments. Significant differences between unbuffered and pH 5.5 samples are indicated by an asterisk (*P* < 0.05).

In fact, PdeN was found to be active during macrocolony biofilm growth specifically under acidic conditions, even when expressed from the single chromosomal copy. Macrocolonies from derivatives of *E. coli* K-12 AR3110 with deletions either in the chromosomal copy of *pdeN* alone or simultaneously in the genes for all five CSS-domain PDEs (∆5CSS) exhibited no significant colony phenotype on standard medium (Fig. 7A; note that these plates do not contain Congo red, which results in a phenotype somewhat different from that in Fig. 1; here, the very flat large colonies with a few highly folded ridges indicate high colony stiffness due to very high matrix production, whereas more compact wrinkly colonies indicate reduced stiffness, that is, somewhat less matrix production). However, when the pH of the growth medium was adjusted to 5.5 by adding N-morpholino-ethane sulfonate (MES), wild-type macrocolonies were smaller and more compact and exhibited only tiny wrinkles, indicating a strong reduction of extracellular matrix production. In the *pdeN* mutant, this effect was less pronounced, suggesting that PdeN is active and contributes to this matrix reduction at low pH. The ∆5CSS mutant missing all five CSS domain PDEs shows a similar phenotype as the *pdeN* deletion alone, indicating that the other four CSS domain PDEs are not involved. In addition, while expressing PdeN from pCAB18 had no effect on macrocolony morphology at standard pH, it resulted in the same strongly reduced matrix phenotype at pH 5.5 (Fig. 7B). As shown with the PdeN^C76A^, PdeN^ASS^, and PdeN^AVL^ variants, this phenotype required the integrity of the DSB in the CSS domain and the active center of the EAL domain, that is, it reflects c-di-GMP degradation by PdeN (Fig. 7B).

To investigate whether the higher activity of PdeN during growth at low pH is linked to increased protein levels, PdeN was expressed chromosomally (i.e., from its natural promoter) with a C-terminal FLAG-tag, and protein levels were compared during growth in liquid media at various pH. Notably, protein amounts of PdeN were elevated at pH 5.5 during the exponential phase and decreased somewhat in samples taken during entry into stationary phase and overnight (Fig. 7C), consistent with *pdeN* (still called *rtn* by reference 30) being expressed from a vegetative promoter in growing cells only (12, 30). Contrary to that, lower basal protein levels throughout the growth cycle were observed at physiological pH, under alkaline conditions (pH 8) as well as in non-buffered medium (Fig. 7C). These results point toward PdeN induction during growth in an acidic environment.

When PdeN^WT^, the DSB-free PdeN^C76A^, and PdeN^ASS^ variants and the enzymatically inactive PdeN^AVL^ were expressed with C-terminal His-tags from the low-copy-number plasmid pCAB18, these proteins were barely detectable in overnight samples when expressed from the leaky p*_tac_* promoter without IPTG induction (Fig. 7D). However, adjusting the pH to 5.5 by adding MES during exponential growth (at an OD_578_ of 0.5) led to significantly higher protein amounts of all these PdeN variants. This not only confirmed induction by a shift to acidic pH but also showed that this induction is post-transcriptional since the proteins were expressed here from an ectopic promoter (p*_tac_*) and independently of redox status and the DSB-dependent structure or activity of PdeN. Strong transcriptional induction by adding IPTG could essentially overrun the more moderate acidic induction of all PdeN variants (Fig. 7D). In the case of the DSB-free variants (PdeN^C76S^ and PdeN^ASS^), IPTG induction also revealed the DegP/Q-dependent degradation fragments as well as larger bands that represent dimers generated by intermolecular DSB formation from the single cysteine still present in these mutant versions of PdeN (Fig. 7D), as previously also described for corresponding PdeC variants (15). The fact that the acidic induction of PdeN has functional consequences for the production of matrix components can be seen not only by qualitatively assessing macrocolony biofilm morphology (Fig. 7A) but also by quantitatively assessing the strongly reduced expression of the c-di-GMP-activated operon encoding the curli fiber subunits (*csgBAC*; assayed using a single-copy *csgB::lacZ* reporter fusion), which was completely dependent on the PDE activity of PdeN, as this effect was not observed with the inactive PdeN^AVL^ variant (Fig. 7E). This experiment also revealed that the plasmid-expressed (i.e., overproduced) DSB-free versions of PdeN retain residual PDE activity—at least upon further induction at low pH, they could still reduce *csgB::lacZ* expression, although less efficiently than PdeN^wt^.

## DISCUSSION

### CSS domain PDEs as model systems for transmembrane signaling

Transmembrane signaling is absolutely crucial for bacteria to sense and appropriately respond to their constantly changing environment. For decades, the molecular mechanisms of information transfer across the cytoplasmic membrane have been investigated with two types of model sensory proteins, that is, chemoreceptors involved in chemotaxis (31) and sensor histidine kinases of two-component signaling systems (32, 33). However, these systems have turned out to be exceedingly complex. Thus, chemoreceptors form large heterogeneous receptor “carpets” with adaptive sensitivity and interact with entire arrays of additional signal-transducing proteins (34, 35). While sensor kinases tend to be smaller, they often integrate several signals on both sides of the membrane, which activates either autophosphorylation or autophosphatase activity, with the first of these activities allowing phosphotransfer and thus information transfer onto at least one other protein, that is, a response regulator, in a core signaling pathway (33).

These complexities contrast with the quite simple structures of membrane-located bacterial enzymes that synthesize or degrade the second messenger c-di-GMP. These DGCs or PDEs often consist of just one periplasmic sensory domain linked by a short TM region to an enzyme domain located on the inner side of the membrane (9). With this minimal domain architecture (Fig. 4) and the active form being just a dimer (15), not in need of interacting with any other protein to show activity (i.e., c-di-GMP hydrolysis), our CSS domain PDEs investigated here are excellent model systems for studying transmembrane signaling. This minimality allows us to easily pinpoint functionally important features such as single or small patches of amino acids required for signaling (Fig. 4A and 5A). Also, purification and reconstitution of activity *in vitro* (in nanodiscs; Fig. 6) are relatively straightforward. In addition, CSS domain PDEs (of the “PdeB type”) use a clear structure-impacting sensing mechanism, that is, the redox transition between free-thiol and DSB forms of the CSS domain, which can be probed genetically (by point mutation; Fig. 1 and 2A) as well as biochemically (by adding DTT; Fig. 2B) and which provides the additional advantage of being detectable by SDS-PAGE/immunoblotting, since only one of the two forms gets processed *in vivo* to a smaller protein fragment by the periplasmic proteases DegP/DegQ (Fig. 3) (15). Finally, *E. coli* K-12 provides us with as many as five CSS domain PDEs (14), which allows for comparative analyses to identify common general principles as well as individual evolutionary adaptations of information processing by these enzymes.

### Redox control of CSS domain PDEs of the “PdeB type”

Structure predictions using AlphaFold2 yield similar structures for all five CSS domain PDEs, although the only obviously common elements in their primary sequences are the conserved domain lengths and a few highly conserved key amino acids, while the sequences between the conserved elements are quite divergent. The N-terminal alpha-helical TM1 continues into a long alpha-helix that serves as an apparently rigid scaffold element that aligns the following two similar globular subdomains that connect to TM2, followed by a canonical EAL domain (Fig. 5). The common structure of all five CSS domain PDEs with a few small but crucial functional elements points toward a shared signaling mechanism for the PDEs of the “PdeB type” that can undergo regulatory DSB/free-thiol transitions as previously elucidated with PdeC (15): the redox state of the CSS domain influences the conformation of the periplasmic CSS domain in a manner that, via the TM2 region, can influence dimerization, which is required for PDE activity of the EAL domain. The crucial DSB is located at the very tip of the CSS domain, pointing into the periplasmic space and compacting the first of the small globular subdomains (Fig. 5). It is readily conceivable that an opening of the DSB relaxes a mechanical constraint that can be transduced via the long alpha helix that includes TM1, which—within the membrane—interacts with TM2 and/or via the second small globular subdomain connected directly to TM2. This relaxation at the tip of the CSS domains may thus lead to a structural flexibilization allowing a slight but crucial rearrangement of the entire PdeB dimer, which, in turn, could stabilize the active EAL domain dimer (Fig. 4D).

### CSS domain PDEs fall into two groups with similar structure and basic biochemistry but opposite effects on enzymatic activity: “PdeB type” vs “PdeN type”

Our comparative analysis of all five CSS domain PDEs of *E. coli* K-12 revealed that the periplasmic redox-dependence of cytoplasmic PDE activity comes in two flavors—besides the “PdeB type” enzymes (PdeB, PdeC, and PdeG) discussed above, which become more active when the DSB is reduced to free thiols (Fig. 1), there is a “PdeN type” (which also seems to include PdeD) that is active in the DSB form. PdeB and PdeN were chosen as experimental prototypes representative of the two classes because their activities *in vivo* are in a range where the different activities of wild-type and the DSB-free ASS mutant variants are clearly shown experimentally (Fig. 1), whereas PdeD and PdeG have very low activities only, while PdeC tends to be overactive when expressed from our low-copy-number plasmid, that is, phenotypic differences are harder to see.

PdeN shows *similar* redox biochemistry (i.e., DSB formation in the CSS domain; Fig. 1 and 2), proteolytic sensitivity (of the free thiol form; Fig. 3), and a crucial role of the TM2 for enzymatic activation (Fig. 1) as PdeB. However, the outcome with respect to PDE activity is fully opposite, that is, PdeN is enzymatically active with its CSS domain in the oxidized DSB form (Fig. 1). In fact, the DSB of PdeN is highly stable—even upon DTT addition to the cells, we could not detect the typical degradation fragment generated by DegP/DegQ that was obtained from the DSB-free PdeN^ASS^ variant as well as from the free-thiol forms of the “PdeB type” CSS domain PDEs (obtained either by DTT treatment or with the ASS variants; Fig. 1 and 2). Also, DSB formation in PdeN was not dependent on the DsbA/DsbB system (Fig. 3). These features are characteristic of a highly stable structural DSB, in contrast to a redox-reversible regulatory DSB (19) as found in the “PdeB type” enzymes.

In addition, our data also revealed that the periplasmic DSB is not the only structural element crucially affecting the enzymatic activities of PdeB and PdeN. In addition, we identified two short amino acid sequence regions termed “box I” and “box II”—connecting the periplasmic CSS domain to TM1 and TM2, respectively—that feature characteristic charge distributions that are not only uncommon for transmembrane proteins—as they violate the “positive-inside rule” (20)—but may also allow electrostatic boxI-boxII interactions that possibly contribute to structural stabilization. Mutating either one or both of these boxes clearly affects the PDE activities of PdeB as well as of PdeN—but again in an opposite manner, that is, PdeB becomes more active, while PdeN is inactivated (Fig. 4C). Thus, these possibly interacting boxI/boxII elements seem to operate along with DSB formation to generate a very constrained or rigid conformation of the periplasmic and transmembrane parts of the proteins, which prevents enzymatic activity for PdeB but represents the active conformation for PdeN, that is, the one that affords dimerization of the cytoplasmic EAL domain.

In PdeN, this rigid and active conformation is further stabilized by additional intramolecular interactions: (i) a unique array of four glycines (4G) within TM2, that is, this helix is slightly kinked and thus not a standard alpha helix, and (ii) a pair of cysteines (C16–C254) positioned to allow the formation of an extra DSB linking TM1/TM2, which is thus located within the transmembrane part of PdeN. Introducing specific point mutations into PdeN showed that both elements contribute to stabilizing the active conformation (Fig. 5), again indicating that activity of PdeN requires a highly constrained rigid structure. The 4G array in TM2 is specific to PdeN—all other CSS domain PDEs have only one or two glycines in TM2. Two cysteines that may form a TM1/TM2-linking DSB are also present in PdeG—whether this element plays a role in PdeG and, if so, which one, remains to be determined.

We conclude that despite considerable sequence divergence among CSS domain PDEs, these proteins contain several distinct small features that impose structural constraints crucial for transmembrane signaling between the periplasmic sensory CSS domain and the cytoplasmic enzymatic EAL domain: (i) the strictly conserved periplasmic DSB, (ii) specifically charged and potentially interacting linkers (box I/box II) between the periplasmic CSS domain and the two flanking TM regions, (iii) glycine kinks in helix TM2, which is crucial for dimerization and thus activation of the EAL domain, and (iv) an optional additional DSB within the transmembrane part (linking TM1 and TM2) of the protein. While the conserved periplasmic DSB (and thus redox sensing in the case of “PdeB type” enzymes) seems the major determinant of enzymatic activity of CSS domain PDEs, these additional elements might also serve as putative targets for additional signal input from as yet unidentified environmental cues.

Although CSS domain PDEs seem to represent the minimal version of a transmembrane-regulated enzyme, evolution has not only created a high informational density in the protein structure but has even managed to generate two types of these enzymes with opposite activation by playing with these small but decisive elements. Thus, PdeN is active in a highly stable and structurally constrained conformation, with mutations in any of these structure-stabilizing elements having an inactivating effect. By contrast, PdeB activation requires structural relaxation, which can be promoted by mutations in any of its structure-stabilizing elements.

### PdeN shows post-transcriptional regulation of expression and a physiological role specifically under acidic growth conditions

The high stability of its structural periplasmic DSB also implies that PdeN—in contrast to the “PdeB-type” CSS domain PDEs—is not subject to redox regulation via DSB/free-thiol transitions and thus could be constitutively active. Hence, its regulation may rather occur at the level of expression, with two observations indicating post-transcriptional regulation: (i) while transcription of the chromosomal copy of *pdeN* (formerly *rtn*) seemed higher than that of *pdeB* (*ylaB*) or *pdeC* (*yjcC*) (30), the actual cellular protein levels of PdeN are in the same range or lower than those of PdeB or PdeC, respectively (12), and (ii) in this study, we had to add inducer (IPTG) to see effects of PdeN and detect the protein when expressed under the control of the ectopic *tac* promoter from a plasmid, whereas PdeB and PdeC did not require induction from similar constructs in order to show activity and be detectable *in vivo* by immunoblotting (Fig. 1 and 2).

This prompted us to search for specific growth conditions under which PdeN might play a phenotypically detectable role and at the same time show post-transcriptional induction. Indeed, we found PdeN to reduce biofilm formation under acidic growth conditions as detectable directly by less macrocolony wrinkling as well as by lower expression of the c-di-GMP-controlled curli operon; consistently, this activity correlated with higher levels of expression of PdeN, no matter whether expressed from its native chromosomal or the ectopic *tac* promoter (Fig. 7), that is, this induction is post-transcriptional.

While investigating the molecular mechanism underlying this post-transcriptional upregulation of PdeN at low pH was beyond the scope of our study presented here, this may be an interesting question for future studies. In principle, this control could act at the levels of the *pdeN* mRNA (e.g., via an acid-inducible sRNA that activates translational initiation) or of the PdeN protein (e.g., by affecting turnover by proteolysis). In comparison to the other CSS domain PDEs, PdeN has a relatively long polar N-terminal sequence preceding TM1 (11 amino acids, corresponding to 33 nucleotides at the mRNA level; Fig. S2). This region may include a target site for translational control on the mRNA (36, 37) or a proteolytic recognition site for a particular protease close to the N-terminus (38). Similar mechanisms of RNA-based as well as proteolysis-mediated controls have been observed for other signal-integrating regulatory proteins, most prominently for the general stress sigma subunit of RNA polymerase, RpoS (σ^S^) (39–41). N-end rule proteolysis mediated by ClpSAP (42–44) might also play a role for PdeN, since it features a destabilizing phenylalanine after its initial methionine (Fig. S2).

### A general and complex role of proteolysis in controlling CSS domain PDEs

All five CSS domain PDEs of *E. coli* are highly regulated, which includes a differential dynamic control of expression as well as of degradation. While the chromosomal *pdeN* gene is expressed from a vegetative RpoD(σ^70^)-dependent promoter during the post-exponential growth phase, the other four genes are activated somewhat later during transition into stationary phase by RpoS(σ^S^)-containing RNA polymerase (12, 30). In addition, all five CSS domain PDEs are substrates for various proteolytic degradation pathways. As discussed above, the post-transcriptional induction of PdeN at low pH is a special case that may include a proteolytic control using a mechanism yet to be determined. On the other hand, PdeN’s rigidly folded periplasmic domain—stabilized by even two DSBs (one involving the CSS element, the other linking TM1 and TM2 within the membrane)—is never attacked by the periplasmic proteases DegP/DegQ, which can also explain its persistent presence even during long-term stationary phase (Fig. 2 and 3), where it is no longer expressed.

This is in pronounced contrast to the other four CSS domain PDEs studied here that are subject to turnover by a common degradation pathway. Once the periplasmic DSB of these PDEs has been eliminated—either naturally by more reducing conditions or experimentally in ASS mutant variants or by DTT treatment, which results in enzymatic activation for PdeB, PdeC, and PdeG—they become more or less efficient substrates for the periplasmic DegP/DegQ protease-mediated protein quality control system (Fig. 3), which recognizes partially unfolded proteins in the periplasm (45–47). As previously shown for PdeC, this allows for two possible inactivation pathways of the enzymatically active, not yet processed free-thiol form (15). Either, DsbA/DsbB-promoted restoration of the periplasmic DSB will drive folding back into an inactive, more compact, again protease-resistant and therefore stably persisting form. Alternatively, the irreversible DegP/DegQ-mediated removal of most of the periplasmic domain—resulting in protein fragments just slightly larger than the genetically constructed TM2-EAL fragments (Fig. 2)—is the first step toward full protein degradation, which involves additional not yet identified membrane-associated and/or cytoplasmic proteases (15). Interestingly, the N-terminally processed fragments are still enzymatically active, as inferred from the activities of the very similar TM2-EAL fragments (except in the case of PdeD; Fig. 1).

Overall, the specific rates—by which the structurally more open free-thiol forms of the periplasmic CSS domains either re-oxidate to form the DSB and structurally refold or are recognized and processed by DegP/DegQ and the follow-up proteases—could be different for the various CSS domain PDEs. Thus, in addition to differential transcriptional and post-transcriptional control of expression and/or redox control of enzymatic activity, also differential fine-tuning of the proteolytic turnover of CSS domain proteins opens opportunities for evolutionary adaptation to specific niches. Given their ability to respond to changes in redox conditions and/or pH, CSS domain PDEs are likely to play a role in oxygen-poor and/or acidic environments, such as in deeper layers of dense biofilms (48), where facultative anaerobic bacteria like *E. coli* operate by acidogenic fermentative energy metabolism. Activation of CSS domain PDEs under these conditions is consistent with the previous observation of denser matrix structures deep within biofilms of *E. coli* mutants lacking all five CSS domain PDEs (15). In addition, such conditions are encountered during the transition of *E. coli* from an outside environment to the gastrointestinal tract. In avian pathogenic *E. coli* (APEC), PdeN was recently observed to promote adhesion and infection by reducing the production of curli fibers and pEtN-cellulose, possibly to expose adhesive fimbriae (49).

## MATERIALS AND METHODS

### Bacterial strains and growth conditions

All strains used are derivatives of the *E. coli* K-12 strain W3110 (50) or AR3110, which is a direct derivative of W3110, in which codon 6 (the stop codon TAG) in the chromosomal copy of *bcsQ* was changed to the sense codon TTG (4). Knockout mutants of *pdeB*(*ylaB*)::*cat*, *pdeC*(*yjcC*)::*kan*, *pdeD*(*yoaD*)::*cat*, *pdeG*(*ycgG*)::*kan*, *pdeH*(*yhjH*)::*kan,* and *pdeN*(*rtn*)::*kan* either alone (30) or in combination in the Δ5CSS mutant (15); *degP::kan*, *degQ::kan*, *dsbA::kan*, and *dsbB::kan* (15); and Δ(*lacI-A*)::*kan* (51) were previously described and were generated as a full open reading frame (ORF) deletions/insertions of antibiotic resistance cassettes using the protocol of one-step-inactivation (52). Mutations associated with antibiotic resistance cassettes were transferred by P1 transduction (53); if required, cassettes were flipped out (52), and the mutations were designated “::*scar.*” C-terminal chromosomal 3× FLAG-tags were added with a two-step method similar to the one-step-inactivation protocol (54). All oligonucleotides used in this study are listed in Table S1. *E. coli* BL21 (DE3) (Novagene) (55) was used for the overexpression and purification of proteins.

If not stated otherwise, strains were cultured in Luria-Bertani (LB) broth (1% tryptone, 0.5% NaCl, and 0.5% yeast extract) with aeration or on LB broth agar plates at 28°C or 37°C. If required, antibiotics or other supplements were used in the following final concentrations: ampicillin (Ap) 100 μg/mL, isopropyl-β-thiogalactopyranoside (IPTG) 0.1 mM, dithiothreitol (DTT) 5 mM, N-morpholino-ethane sulfonate (MES; pH 5.5), and 100 mM N-morpholino-propane sulfonate (MOPS; pH 7.0 or 8.0).

### Construction of low-copy-number plasmids expressing wild-type and mutant variants of all five CSS domain phosphodiesterases

The plasmids used in this study were constructed using the low-copy-number vector pCAB18, which carries the *tac* promoter, followed by a multiple cloning site (56). C-terminal His6-tags fused to the CSS domain PDE variants were introduced via the oligonucleotide primers used for PCR (Table S1). Point mutants harboring an amino acid exchange of PDE variants were generated by splicing by overlap extension (SOE) (57). Four primers were used, with two introducing the mutation and the others flanking the up- and downstream regions of the gene and carrying restriction sites for cloning into pCAB18. In the first step, two separate PCR fragments were generated that overlap in the region of the mutation and cover the up- and downstream regions, respectively. In the second step, these fragments were used in combination as templates for a PCR reaction with the up- and downstream primers only to generate the final PCR fragment. This fragment contains the entire gene carrying the mutation flanked by the restriction sites for cloning, which was then inserted into the vector.

### Growth of bacterial macrocolony biofilms

Cells were grown overnight in liquid LB medium (53) under aeration at 37°C; 5 µL of overnight cultures were spotted on salt-free LB agar plates supplemented with Congo Red (CR; 40 µg/mL) and Coomassie Brilliant Blue (20 µg/mL). For pH experiments, salt-free LB plates were either adjusted to pH 5.5 (with MES) or left unbuffered without adding CR, respectively. Plates were incubated at 28°C for 5 days.

### Stereomicroscopy

*E. coli* macrocolony biofilms were visualized at 10× magnification with a Stemi 2000-C stereomicroscope (Zeiss, Oberkochen, Germany). Digital images were captured with an AxioCam ICC3 digital camera coupled to the stereomicroscope, operated via the AxioVision 4.8 software (Zeiss).

### Determination of β-galactosidase (LacZ) activity

β-Galactosidase assays were carried out with strains harboring a single-copy *csgB::lacZ* reporter fusion (58) inserted at the *att*(λ) site in a Δ(*lacI-A*)::scar background. Enzymatic activities were assayed by the use of *o*-nitrophenyl-β-D-galactopyranoside (ONPG) as a substrate and are reported as µmol of *o*-nitrophenol per min per mg of cellular protein (53). Experiments were performed in three or more biological replicates.

### SDS-PAGE and immunoblot analysis

For immunoblot analyses, samples were taken in the exponential (OD_578_ of 0.5) and postexponential (OD_578_ of 1.0, 2.0, or 3.0) growth phases and/or from overnight cultures grown in LB medium. Samples corresponding to 120 µg of total cellular protein were precipitated with 10% trichloroacetic acid (TCA). The protein pellets were resuspended in SDS-PAGE sample buffer without DTT and incubated for 10 min at 70°C and 95°C, respectively. Samples were run on 12% SDS-polyacrylamide gels. In cases where the apparent sizes of proteins had to be compared, the total protein content of the samples was adjusted to yield similar amounts of the relevant proteins. Proteins were detected by immunoblotting as previously described (59) using antibodies against His6-tag (Bethyl Laboratories, Inc.) or Flag tag (Sigma). Anti-rabbit IgG horseradish-peroxidase conjugate from donkey (GE Healthcare) was used for protein visualization in the presence of Western Lightning Plus-ECL enhanced chemiluminescence substrate (PerkinElmer). Standard protein size markers were obtained from Thermo Scientific.

### Overproduction and purification of variants of the CSS domain PDEs

C-terminally His6-tagged versions of PdeB^C285S^ and PdeN^C29S^ were purified from pCAB18-derived plasmids in the *E. coli* BL21 (DE3) strain. Cultures were grown in LB with aeration at 37°C to an OD_578_ of 0.5–0.7 following induction with IPTG (0.1 mM) for 4 h at 37°C. Cells were harvested and lysed by three passages through a French press. Membrane proteins were purified essentially as described (15, 60). Cell debris was pelleted by centrifugation at 5,000 rpm for 20 min. Membranes were pelleted by ultracentrifugation at 36,000 rpm for 120 min, resuspended and homogenized in 50 mM Tris-HCl (pH 8), 10 mM MgCl_2_, and 300 mM NaCl with protease inhibitor (cOmplete Tablets, Roche), and solubilized in the presence of 2% (wt/vol) n-dodecyl β-D-maltoside (DDM; Sigma Aldrich) and 5% (vol/vol) glycerol under agitation at 4°C for 2 h. Non-solubilized material was pelleted by ultracentrifugation for 1 h at 36,000 rpm. The supernatant containing the solubilized membrane fraction was used for protein purification. Imidazole was added to a final concentration of 20 mM, and the solution was incubated with Ni-NTA (Protiono Ni-NTA Agarose, Macherey-Nagel) overnight. The whole solution was transferred onto chromatography columns (Bio-Rad) and washed with 50 mM Tris-HCl (pH 8) containing 0.05% DDM, 5% glycerol, 300 mM NaCl, 10 mM MgCl_2_, protease inhibitor, and imidazole up to 20 mM. Proteins were eluted by the addition of 250 mM imidazole. For the reconstitution into nanodiscs, the buffer was exchanged to 20 mM Tris-HCl (pH 7.5), 10 mM MgCl_2_, 100 mM NaCl, and 3% glycerol using PD-10 columns (Cytiva). Purified protein was concentrated by Amicon Ultra-4 centrifugal filters (30,000 NMWL; Merck Millipore), and protein concentrations were measured using Nanodrop 2000 (PEQLAB, Biotechnologie-GmbH). Samples were stored at −80°C.

### Reconstitution of CSS domain PDE variants and PDE activity assays in lipid bilayer nanodiscs

For the reconstitution of His6-tagged PDE variants into nanodiscs (28), we followed previously published protocols (29, 61), with some variations; 12.5 mg of chloroform-solubilized *E. coli* total lipid extract (Avanti Polar Lipids) was vacuum-dried. Lipids were subsequently hydrated by the addition of nanodisc (ND) buffer (20 mM Tris [pH 7.5], 100 mM NaCl) and DDM (0.01% final concentration), followed by 5–10 min of sonication. Purified membrane scaffold protein MSP1E3D1 and purified PDE variants were added to the solubilized lipids in equimolar ratios of 1:5:1,200 between PDE variants, the scaffold protein, and the lipids. Samples were incubated for 1 h at 4°C, and the detergent was removed using 1.5 g wet Bio-Beads SM-2 (Bio-Rad, equilibrated in ND buffer) per 5 mL volume. To prevent lipid oxidation, the samples were covered with N_2_ gas. After 5 h of incubation at 4°C, ND buffer containing the nanodiscs was separated from the beads and incubated with Ni-NTA agarose (Protiono Ni-NTA Agarose, Macherey-Nagel) overnight. Purification of nanodiscs with incorporated PDE variants was performed as described (29, 61), but without detergent and protease inhibitor. Purified nanodiscs were concentrated by Amicon Ultra-4 centrifugal filters (10,000 NMWL; Merck Millipore). Relative approximate concentrations of the scaffold protein and PDE proteins in the nanodiscs were controlled by SDS polyacrylamide gel electrophoresis with marker proteins at standardized concentrations (Amersham Low Molecular Weight Calibration Kit for SDS Electrophoresis, GE Healthcare). Nanodisc preparations were stored at 4°C for no longer than 7 days.

### Phosphodiesterase activity assay

PDE activity of PdeB and PdeN variants reconstituted in nanodiscs was performed as described (62) with [α-^32^P]c-di-GMP (Hartmann Analytic) as a substrate. Protein concentration in the assay was 57 nM, and 5 mM DTT was added to the reactions, where indicated. Samples were taken at different times up to 90 min (as mentioned in the figures) and were analyzed by thin-layer chromatography and phosphoimaging (Typhoon FLA-7000, Fuji). To generate the control spot of GTP on thin-layer chromatographs, [α-^32^P]GTP (Hartmann Analytic) was used.

### Protein structure prediction

The ColabFold v1.5.2 notebook (63) was used to create models of protein monomers and dimers with AlphaFold2 (64). Due to certain limitations of AlphaFold2 with membrane proteins (64), we used a database established especially for transmembrane proteins (TMAlphaFold database), which uses a geometry-based method to visualize the most likely position of the protein within the membrane and allows showing whether the predicted 3D structure is realistic (65). Values obtained for the prediction of the arrangement of the TM helices, their orientation within the lipid bilayer (CCTOP Consensus Constrained TOPology prediction algorithm), and the positions and boundaries of the TM segments (TMDET TransMembrane Domain Extraction Tool) were good for PdeB and excellent for PdeN. Protein sequences were obtained from UniProt with the IDs for each protein listed as follows: PdeB P77473, PdeC P32701, PdeD P76261, PdeG P75995, and PdeN P76446. Molecular graphics and analysis were performed using the software ChimeraX 1.5 (66, 67).

### Statistics

Unless otherwise stated, experiments reported in this study were done in at least three biological replicates. In photographic documentation, representative results are shown. Data were analyzed using GraphPad Prism version 9, and the differences were considered significant at *P*-values of <0.05.

## SUPPLEMENTAL MATERIAL

Supplemental material mbio.01325-26-s0001.pdf Figures S1-S4 and Table S1.

ASM does not own the copyrights to Supplemental Material that may be linked to, or accessed through, an article. The authors have granted ASM a non-exclusive, world-wide license to publish the Supplemental Material files. Please contact the corresponding author directly for reuse.
