# Supplementary material for: Redox control and mechanisms of transmembrane signaling in CSS domain c-di-GMP phosphodiesterases that control biofilm formation in Escherichia coli
Source: mBio. 2026 Sep 1;17(10):e01325-26. doi: 10.1128/mbio.01325-26 (PMC13712737; doi:10.1128/mbio.01325-26)
Supplement: Supplementary file 1 [file mbio.01325-26-s0001.pdf]

# SUPPLEMENTAL MATERIAL

# Redox control and mechanisms of transmembrane signaling in CSS domain c-di-GMP phosphodiesterases that control biofilm formation in *E. coli*

**Katharina Pressler, Martin Lorkowski, and Regine Hengge***

## CONTENTS

### 1. Supplementary Figures


Figure S1 Effect of *degQP* mutation on macrocolony morphology in *E. coli* K-12 AR3110.

Figure S2. AlphaFold2 confidence scores and amino acid sequence alignment of PdeB and PdeN.

Figure S3. Cellular expression levels of PdeB and PdeN variants.

Figure S4. Point mutation in PdeB$^{C285S}$ can slightly reduce the PDE activity of the DSB-free PdeB$^{ASS}$ variant.


### 2. Supplementary Table (Oligonucleotides)

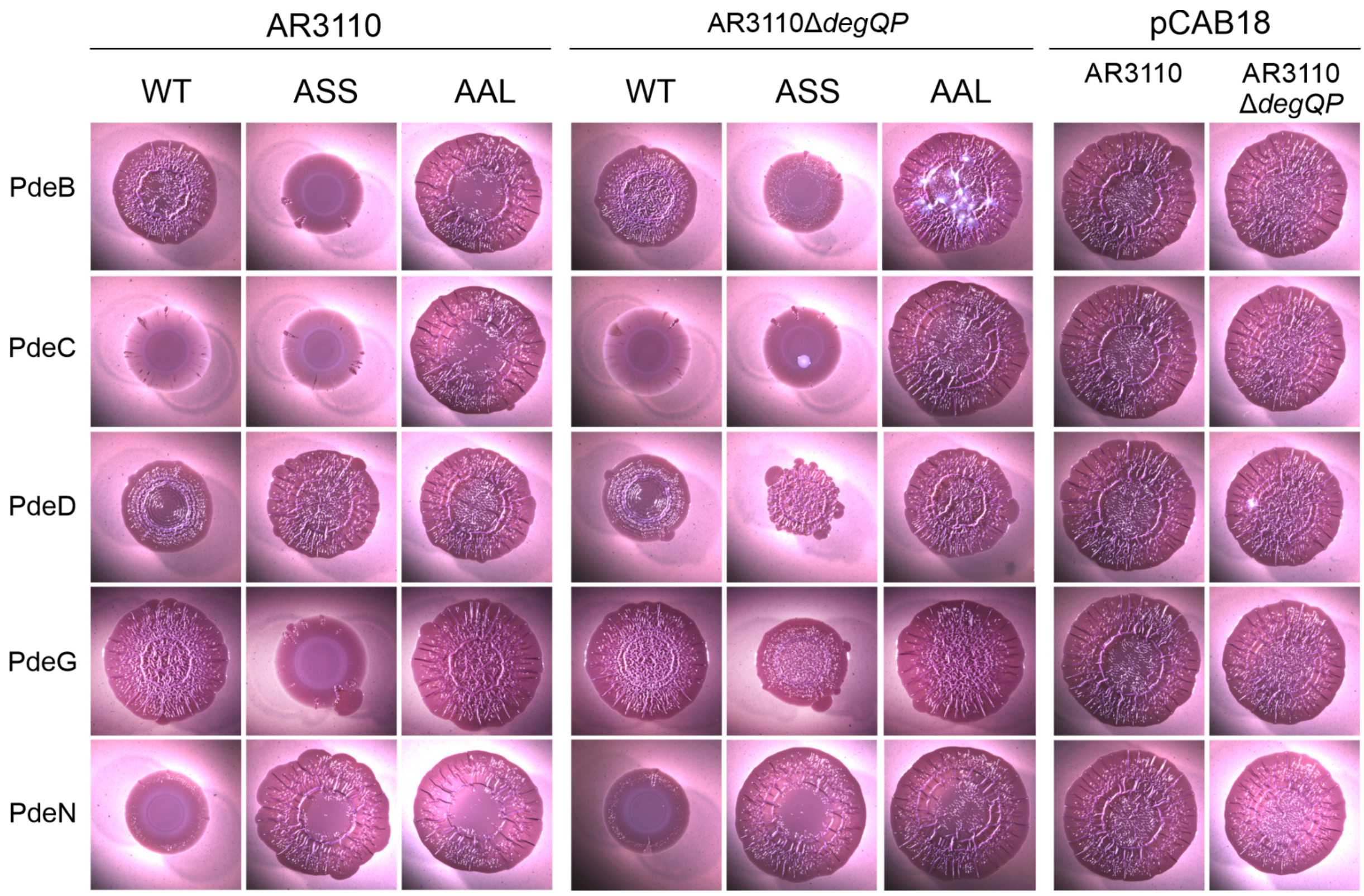


**Fig. S1: Effect of *degQP* mutation on macrocolony morphology in *E. coli* K-12 AR3110.** Macrocolonies of *E. coli* K-12 strain AR3110 and its Δ*degQP* mutant derivative expressing the indicated variants of the five CSS domain PDEs from the low copy number plasmid pCAB18 were grown on salt-free LB agar plates also containing the matrix dye Congo red for 5 days at 28°C. Cultures of strains expressing PdeN variants were additionally induced with 0.1 mM IPTG.

A

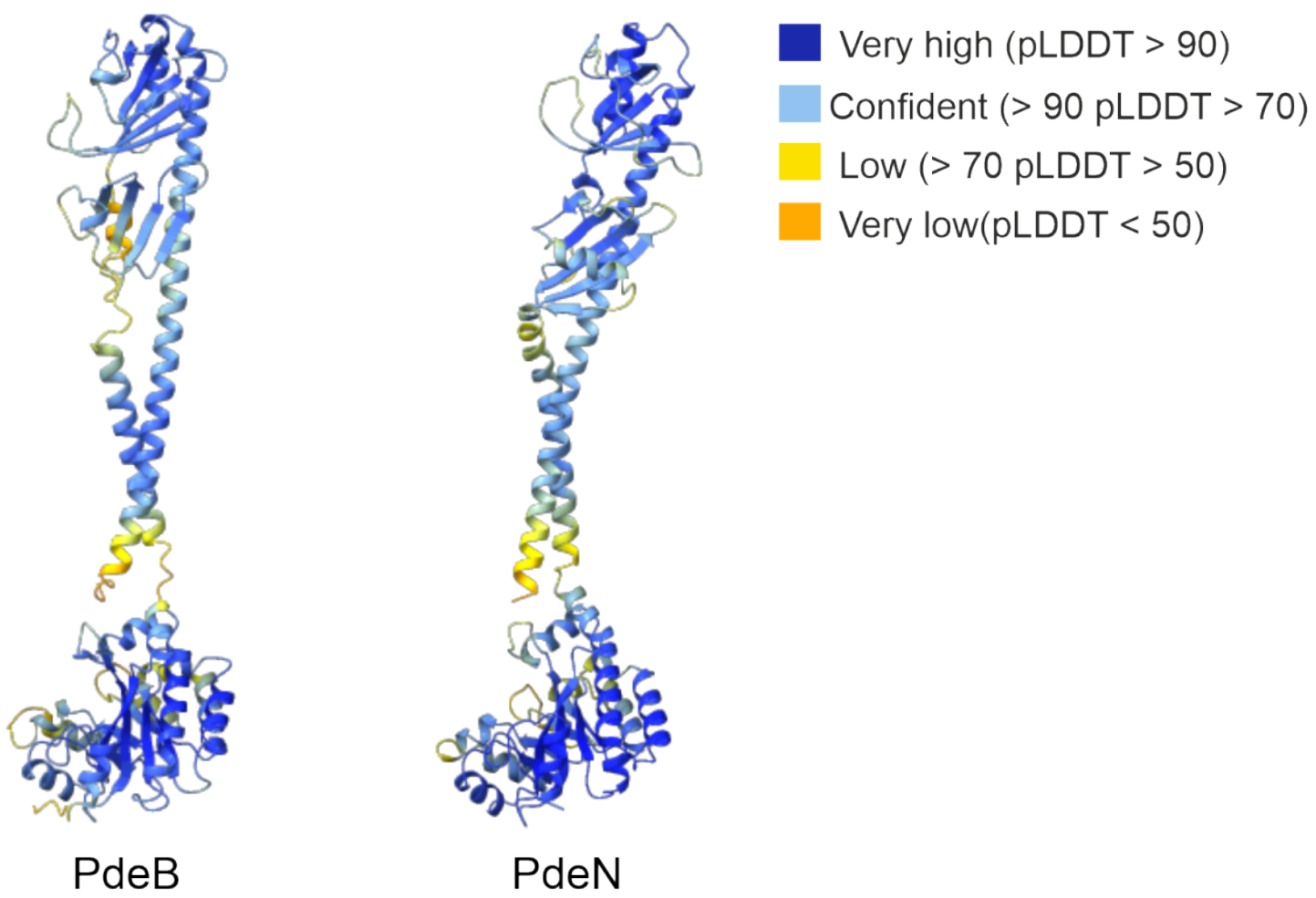


B

```
PdeB  1 MRTRHLVG LISGVLILSV LLPVGLSIWL AHQQVETSFI EELDTYSSRV AIRANKVATQ GKDALQELER 70
PdeN  MFIRAPNFGR KLLLTCIVAG VMIAILVSCL QFLVAWHKHE VKYDTLITDV QKYLDTYFAD LKSTTDRLQP
        :*: ::    :  . *::  :: . *   *  .  .  ..   : **  : *     :.  ::  *.: :.*:
PdeB  WQGAACSEAH LMEMRRVSYS YRYIQEVAYI DNNVPQCSSL EHESPPDTFP EPGKISKDGY RVWLTSHNDL 140
PdeN  LTLDTCQQAN PELTARAAFS MNVRTFVLVK DKKTFCSSAT GEMDIPLNEL IP-------- ALDINKNVDM
          :*.:*:      *.::*  .    *    *::.  .*:   . . * .    *          : :..: *:
PdeB  GIIRYMVAMG TAHYVVMIDP ASFIDVIPYS SWQIDAAIIG NAHNVVITSS DEIAQGIITR LQKTPGEHIE 210
PdeN  AILPGTPMVP NKPAIVIWYR NPLLKNSGVF AALNLNLTPS LFYSSRQEDY DGVALIIGNT ALSTFSSRLM
      .*:     :  .   :*:     .::.      :        .   :.    .  * :*  * .    .* ..::
PdeB  NNGIIYDILP LPEMNISIIT WASTKMLQKG WHRQVFIWLP LGLVIGLLAA MFVLRILRRI QSPHHRLQD  280
PdeN  NVNELTDMPV RETKIAGIPL TVRLYADDWT WN-DVWYAFL LGGMSGTVVG LLCYYLMSVR MRPGREIMT
      * . : *:         .*    .     :   *:  :*:  :  ** : * :.. ::   ::      * :.:
PdeB  AIENRDICVH YQPIVSLANG KIVGAEALAR WPQTDGSWLS PDSFIPLAQQ TGLSEPLTLL IIRSVFEDMG 350
PdeN  AIKREQFYVA YQPVVDTQAL RVTGLEVLLR WRHPVAGEIP PDAFINFAES QKMIVPLTQH LFELIARDAA
      **:..:: *  ***:*.     ::.* *.* * * :. .. :. **:** :*:.   :  ***   ::. : .* .
PdeB  DWLRQHPQQH IS-INLESPV LTSEKIPQLL RDMINHYQVN PRQIALELTE REFADPKTSA PIISRYREAG 420
PdeN  ELEKVLPVGV KFGINIAPDH LHSESFKADI QKLLTSLPAH HFQIVLEITE RDMLKEQEAT QLFAWLHSVG
      :  :  *       **: .   * **.:   : :.::.   .:   **.**:** *:: . : ::  :::  :..*
PdeB  HEIYLDDFG TGYSSLSYLQD LDVDILKIDK SFVDALEYKN VTP----HII EMAKTLKLKM VAEGIETSKQ 490
PdeN  VEIAIDDFG TGHSALIYLER FTLDYLKIDR GFINAIGTET ITSPVLDAVL TLAKRLNMLT VAEGVETPEQ
       ** :**** **:*:* **:  : :* ****: .*::*:  :. :*.     ::  :** *::   ****:**.:*
PdeB  EEWLRQHGV HYGQGWLYSKA LPKEDFLRWA EQHL---- 538
PdeN  ARWLSERGV NFMQGYWISRP LPLDDFVRWL KKPYTPQW
       .** ::** :: **:  *:. ** :**:**  ::
```

**Fig. S2: AlphaFold2 confidence scores and amino acid sequence alignment of PdeB and PdeN.** PdeB[WT] (Uniprot ID P77473) and PdeN[WT] (Uniprot ID P76446) was modeled using AlphaFold2 and depicted with ChimeraX 1.5. Each residue in the sequence is color-coded based on the model confidence score, pLDDT, as indicated. (B) Amino acid sequences of PdeB and PdeN were aligned using the Clustal Omega multiple sequence alignment tool. Symbols below each position in the alignment refer to the conservation score and are indicated as follows: conserved (*), conservative mutation (:), semi-conservative mutation (.), non-conservative mutation ( ) and gap (-). TM regions of both proteins are highlighted in green, and the highly charged regions directly following TM1 (boxI) and just preceeding TM2 (boxII) are colored according to their charge (blue indicates amino acids with positive charge and red indicates those with negative charge). The conserved CSS motif in the periplasmic loop is marked in yellow, and the characteristic EA(V)L motif in the C-terminal cytoplasmic domain is highlighted in grey.

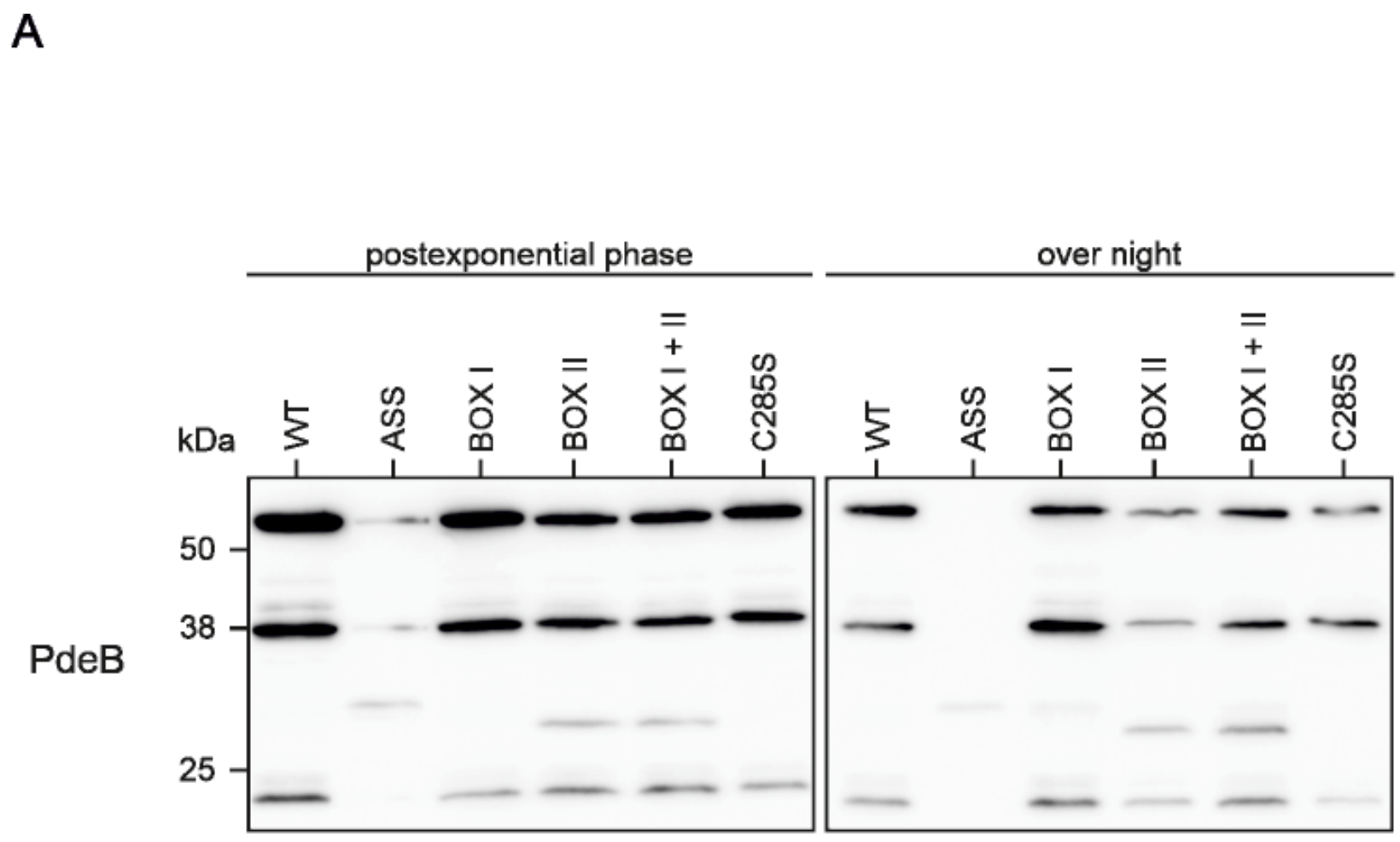


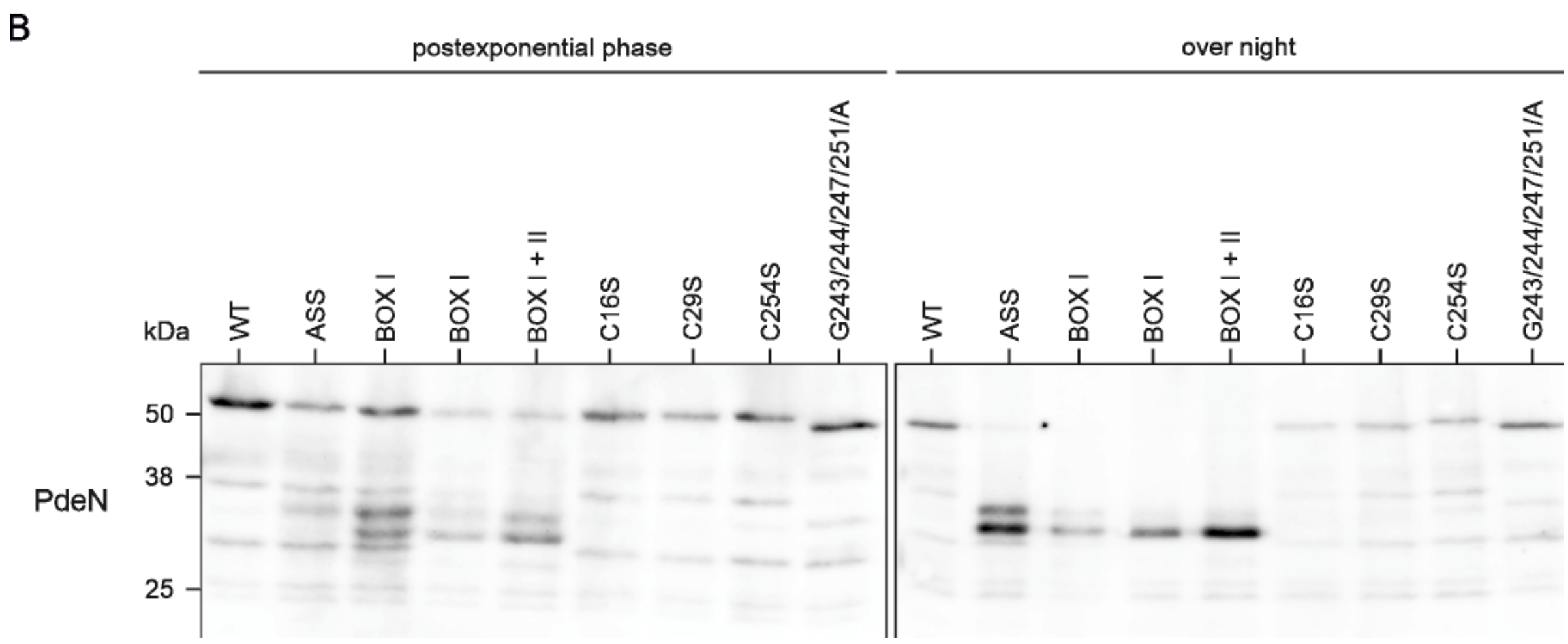


**Fig. S3: Cellular expression levels of PdeB and PdeN variants.** Derivatives of strain W3110 expressing the indicated PdeB and PdeN variants, which harbor a C-terminal His6-tag, from pCAB18 were grown at 28°C in salt-free LB (with 0.1 mM IPTG added for PdeN variants). For the nomenclature of the 'BOX' variants, see legend to main Fig. 4. Samples were taken during post-exponential ($OD_{578}$ approx. 2.5) and after overnight growth and the PdeB and PdeN variants were visualized by SDS-PAGE and immunoblotting using antibodies against the 3xFLAG-tag.

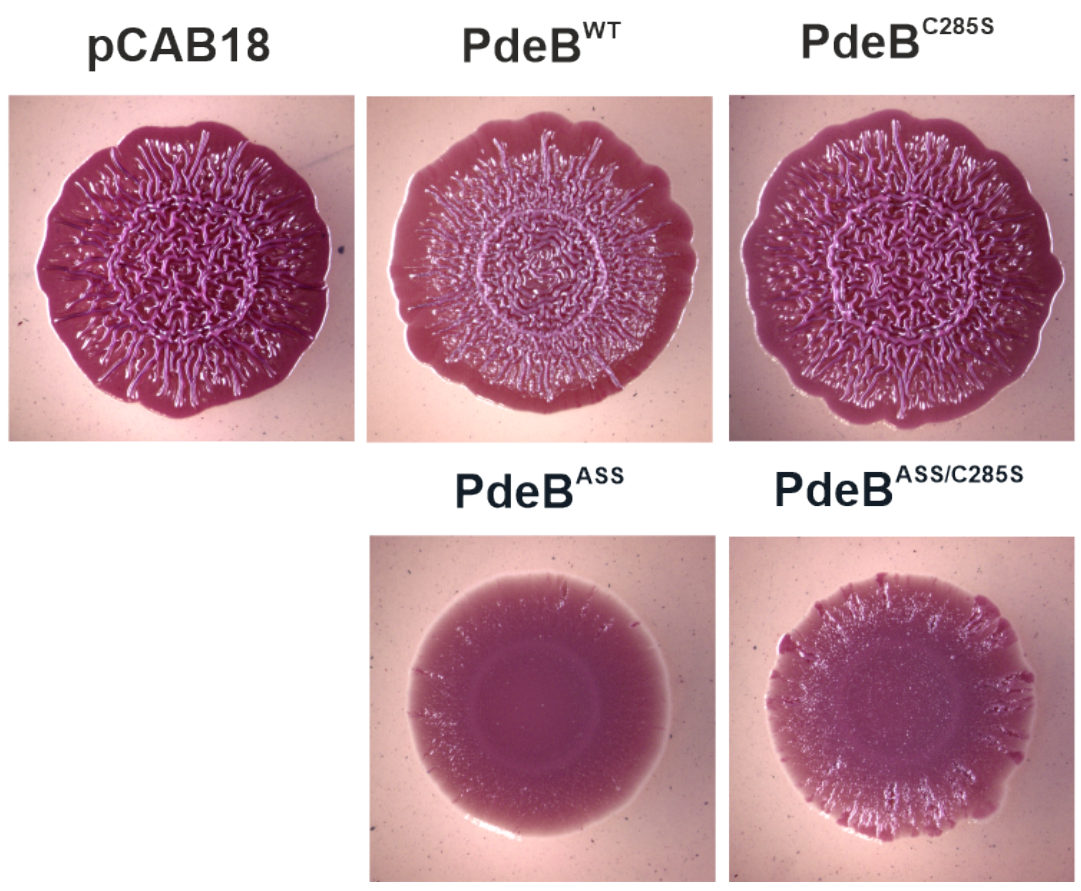


**Fig. S4: Point mutation in PdeB$^{C285S}$ can slightly reduce the PDE activity of the DSB-free PdeB$^{ASS}$ variant.** Macrocolonies of strain AR3110 expressing the indicated PdeB variants from the low copy number plasmid pCAB18 were grown on salt-free LB agar plates containing Congo red for 5 days at 28°C.

## Table S1. Oligonucleotides.

I. Primers used for constructing pCAB18 derivatives that express CSS domain PDE variants or fragments with C-terminal 6xHIS-tags [1, 2]

| | |
|---|---|
| *pdeB* EcoRI FW | GAC*GAATTC*ATGCTGGTGAGAACACGACATC |
| *pdeB* SacI REV | GAC*GTCGAC*TCACAAATGTTGCTCGGCCCAGC |
| *pdeB* TM2+EAL EcoRI REV | GAC*GAATTC*ATGCAAGTCTTTATTTGGTTACCGC |
| *pdeB* EAL EcoRI REV | GAC*GAATTC*ATGATTTTGCGCCGTATTCAGTCAC |
| *pdeB* 6xHIS SacI REV | GCA*GAGCTC*TCA**GTGATGGTGATAGTGATG**CAAATGTTGCTCGGCCCA |
| *pdeD* EcoRI FW | GAC*GAATTC*ATGCAAAAAGCACAACGGATC |
| *pdeD* SalI REV | GAC*GTCGAC*TTAACGTAACGGCATAATGGGC |
| *pdeD* TM2+EAL EcoRI FW | GAC*GAATTC*ATGCAATTACCGCTGGCGGTGCTG |
| *pdeD* EAL EcoRI FW | GAC*GAATTC*ATGGCTTACCGAATGAGCTTTTC |
| *pdeD* 6xHIS SalI REV | GAC*GTCGAC*TTA**GTGATGGTGATGGTGATG**ACGTAACGGCATAATGGGC |
| *pdeG* BamHI FW | GAC*GGATCC*ATGCGCAATACACTCATACC |
| *pdeG* SacI REV | GTC*GAGCTC*TCACTCAACCACAACCTTCA |
| *pdeG* TM2+EAL BamHI FW | GAC*GGATCC*ATGGGCTTTGGGATTTTAAT |
| *pdeG* EAL BamHI FW | GAC*GGATCC*ATGGATAGATATTTCAATA |
| *pdeG* 6xHIS SacI REV | GTC*GAGCTC*TCA**GTGATGGTGATGGTGATG**CTCAACCACAACCTTCACC |
| *pdeN* EcoRI FW | GAC*GAATTC*ATGTTCATACGCGCTCCCAAT |
| *pdeN* SalI REV | GAC*GTCGAC*TTACCACTGCGGCGTATACGG |
| *pdeN* TM2+EAL SalI FW | GAC*GAATTC*ATGAACGATGTGTGGTACGCATTTTTAC |
| *pdeN* EAL SalI FW | GAC*GAATTC*ATGCTGATGAGCGTACGTATGCGC |
| *pdeN* 6xHIS SalI REV | GAC*GTCGAC*TTA**GTGATGGTGATGGTGATG**CCACTGCGGCGTATACGG |

Primers used for introducing mutations into CSS domain PDEs expressed from pCAB18 derivatives[1, 2]:

| | |
|---|---|
| *pdeB* C285S REV: | ATAGTGCAC**GGA**AATATCACGATTTTCGATAGC |
| *pdeB* C285S FW: | GATATT**TCC**GTGCACTATCAGCCGATTGTCTCC |
| *pdeN* C16S REV: | GCCTGCAACAAT**GGA**GGTAAGCAGGAGCTTACG |
| *pdeN* C16S FW: | CTTACC**TCC**ATTGTTGCAGGCGTAATGATTGCG |
| *pdeN* C29S REV: | TAAAAACTGAAG**GGA**ACTCACCAGTATCGCAAT |
| *pdeN* C29S FW: | GTGAGT**TCC**CTTCAGTTTTTAGTGGCCTGGCAT |
| *pdeN* C254S REV: | CAGGTAATA**GGA**GAGCAGGCCAACGACAGTTCC |
| *pdeN* C254S FW: | GGCCTGCTC**TCC**TATTACCTGATGAGCGTACGT |
| *pdeB* box I *pdeN* FW | **CATAAGCACGAAGTCAAATACGACACACTGATTACCGAC**ACCTATTCCTCCCGC |
| *pdeB* box I *pdeN* REV | **GTCGGTAATCAGTGTGTCGTATTTGACTTCGTGCTTATG**GGCCAGCCAGATGCTT |
| *pdeB* box II *pdeN* FW | **CGGCTTTATGCAGATGACTGGACATGGAACGAT**GTCTTTATTTGGTTA |
| *pdeB* box II *pdeN* REV | **ATCGTTCCATGTCCAGTCATCTGCATAAAGCCG**CGTTGAAGCCCATGTG |
| *pdeN* box I *pdeB* FW | **CATCAGCAGGTAGAAACATCGTTTATTGAAGAGCTGGAT**GTACAAAAGTATCTCG |
| *pdeN* box I *pdeB* REV | **ATCCAGCTCTTCAATAAACGATGTTTCTACCTGCTGATG**CCAGGCCACTAAAAAC |
| *pdeN* box II *pdeB* FW | **AAAATGTTGCAGAAAGGCTGGCATCGGCAA**GTGTGGTACGCATTT |
| *pdeN* box II *pdeB* REV | **TTGCCGATGCCAGCCTTTCTGCAACATTTT**AACGGTCAGAGGAAT |
| *pdeN* G243/244/247/251A REV | **CGC**AACGACAGT**CGC**ACTCAT**CGCCGC**CAGTAAAAATGCGTA |
| *pdeN* G243/244/247/251A REV | **GCGGCG**ATGAGT**GCG**ACTGTCGTT**GCG**CTGCTCTGCTATTA |
| *pdeB* EVL-AVL FW | TGTCGGTGCT**GCG**GCACTGGCGCGCTGGC |
| *pdeB* EVL-AVL REV | GCCAGCGCGCCAGTGC**CGC**AGCACCGACA |
| *pdeB* CSS-ASS FW | CGTTCCCCAG**GCT**TCGTCTCTGGAGCA |
| *pdeB* CSS-ASS REV | TGCTCCAGAGACGA**AGC**CTGGGGAACG |
| *pdeD* EIL-AIL FW | TATTGGTGTA**GCG**ATTTTGCTGCGCTGGA |
| *pdeD* EIL-AIL REV | TCCAGCGCAGCAAAAT**CGC**TACACCAATA |
| *pdeD* CSS-ASS FW | CACACTTTAT**GCC**TCCAGCATTTTTGGTTA |
| *pdeD* CSS-ASS REV | TAACCAAAAATGCTGGA**GGC**ATAAAGTGTG |
| *pdeG* EVL-AVL FW | GGGAGTT**GCG**GTGTTAGCCCGCT |
| *pdeG* EVL-AVL REV | AGCGGGCTAACAC**CGC**AACTCCC |
| *pdeG* CTS-ATS FW | TAGTCTGG**GCT**ACATCCCTGCCTG |
| *pdeG* CTS-ATS REV | CAGGCAGGGATGT**AGC**CCAGACTA |
| *pdeN* EVL-AVL FW | CGAGTAACGGGCCTG**GCA**GTACTGCTACGCTGG |
| *pdeN* EVL-AVL REV | CCAGCGTAGCAGTACTGCCAGGCCCGTTACTCGC |
| *pdeN* CSS-ASS FW | GATAAAAAAACATTC**GCT**TCATCTGCGACCGGT |
| *pdeN* CSS-ASS REV | ACCGGTCGCAGATGA**AGC**GAATGTTTTTTTATC |
| *pdeN* C76A FW | CCGCTGACCTTAGATACC**GCC**CAGCAAGCTAAC |
| *pdeN* C76A REV | GTTAGCTTGCTG**GGC**GGTATCTAAGGTCAGCGG |

[1] Restriction sites are indicated in *italics*.
[2] Mutations introduced are indicated in **bold**.
